# Inflammation and Disintegration of Intestinal Villi in an Experimental Model for *Vibrio parahaemolyticus*-Induced Diarrhea

**DOI:** 10.1371/journal.ppat.1002593

**Published:** 2012-03-15

**Authors:** Jennifer M. Ritchie, Haopeng Rui, Xiaohui Zhou, Tetsuya Iida, Toshio Kodoma, Susuma Ito, Brigid M. Davis, Roderick T. Bronson, Matthew K. Waldor

**Affiliations:** 1 Brigham and Women's Hospital/Harvard Medical School and HHMI, Boston, Massachusetts, United States of America; 2 Department of Bacterial Infections, International Research Center for Infectious Diseases, Osaka University, Suita, Osaka, Japan; 3 Laboratory of Genomic Research on Pathogenic Bacteria, International Research Center for Infectious Diseases, Osaka University, Suita, Osaka, Japan; 4 Department of Neurobiology, Harvard Medical School, Boston, Massachusetts, United States of America; 5 Department of Microbiology & Immunology, Harvard Medical School, Boston, Massachusetts, United States of America; Duke University, United States of America

## Abstract

*Vibrio parahaemolyticus* is a leading cause of seafood-borne gastroenteritis in many parts of the world, but there is limited knowledge of the pathogenesis of *V. parahaemolyticus*-induced diarrhea. The absence of an oral infection-based small animal model to study *V. parahaemolyticus* intestinal colonization and disease has constrained analyses of the course of infection and the factors that mediate it. Here, we demonstrate that infant rabbits oro-gastrically inoculated with *V. parahaemolyticus* develop severe diarrhea and enteritis, the main clinical and pathologic manifestations of disease in infected individuals. The pathogen principally colonizes the distal small intestine, and this colonization is dependent upon type III secretion system 2. The distal small intestine is also the major site of *V. parahaemolyticus*-induced tissue damage, reduced epithelial barrier function, and inflammation, suggesting that disease in this region of the gastrointestinal tract accounts for most of the diarrhea that accompanies *V. parahaemolyticus* infection. Infection appears to proceed through a characteristic sequence of steps that includes remarkable elongation of microvilli and the formation of *V. parahaemolyticus*-filled cavities within the epithelial surface, and culminates in villus disruption. Both depletion of epithelial cell cytoplasm and epithelial cell extrusion contribute to formation of the cavities in the epithelial surface. *V. parahaemolyticus* also induces proliferation of epithelial cells and recruitment of inflammatory cells, both of which occur before wide-spread damage to the epithelium is evident. Collectively, our findings suggest that *V. parahaemolyticus* damages the host intestine and elicits disease via previously undescribed processes and mechanisms.

## Author Summary

The marine bacterium *Vibrio parahaemolyticus* is a leading cause worldwide of gastroenteritis linked to the consumption of contaminated seafood. Despite the prevalence of *V. parahaemolyticus*-induced gastroenteritis, there is limited understanding of how this pathogen causes disease in the intestine. In part, the paucity of knowledge results from the absence of an oral infection-based animal model of the human disease. We developed a simple oral infection-based infant rabbit model of *V. parahaemolyticus*-induced intestinal pathology and diarrhea. This experimental model enabled us to define several previously unknown but key features of the pathology elicited by this organism. We found that *V. parahaemolyticus* chiefly colonizes the distal small intestine and that the organism's second type III secretion system is essential for colonization. The epithelial surface of the distal small intestine is also the major site of *V. parahaemolyticus*-induced damage, which arises via a characteristic sequence of events culminating in the formation of *V. parahaemolyticus*-filled cavities in the epithelial surface. This experimental model will transform future studies aimed at deciphering the bacterial and host factors/processes that contribute to disease, as well as enable testing of new therapeutics to prevent and/or combat infection.

## Introduction

*Vibrio parahaemolyticus* is a Gram-negative bacterium that resides in the marine environment, often in association with shellfish. It is a leading cause of gastroenteritis linked to consumption of raw or undercooked seafood throughout the world, and especially in Asia [1]. Infections caused by *V. parahaemolyticus* can occur sporadically or in outbreaks, which can be relatively large. For example, in 2005, more than 10,000 people were sickened by *V. parahaemolyticus*-contaminated clams and mussels in Chile [2]. The most common clinical manifestation of *V. parahaemolyticus* infection of the gastrointestinal tract is acute, self-limited watery diarrhea that is often accompanied by abdominal pain, nausea and vomiting. However, the severity of symptoms can range from mild watery diarrhea to a severe dysentery-like illness [3]. *V. parahaemolyticus* has also been linked to wound infections and septicemia; however, such infections are far less common and generally not food-borne.

Despite the prevalence of *V. parahaemolyticus*-induced gastroenteritis, there is limited understanding of the pathogenesis of *V. parahaemolyticus*-induced diarrhea. In part, this paucity of knowledge is explained by the fact that few analyses of intestinal tissue from *V. parahaemolyticus*-infected patients have been performed, few human volunteer studies have been carried out, and there is no oral-infection-based animal model that closely resembles the human disease. However, human studies suggest that *V. parahaemolyticus* infection disrupts the intestinal epithelium. Autopsy findings from the first reported epidemic of *V. parahaemolyticus* in Japan noted ‘slight erosion of the jejunum and ileum’ in several patients who died due to the infection [4]. In the most extensive study to date, rectal and duodenal biopsies from patients with acute *V. parahaemolyticus* infections revealed ‘epithelial degeneration and denudation’ at both sites [5], creating some uncertainty regarding the intestinal site (small or large bowel) and processes that give rise to the watery diarrhea that usually accompanies *V. parahaemolyticus* infection. Additionally, there was evidence of an acute inflammatory response, marked by polymorphonucleocyte (PMN) infiltration at both sites, as well as elevated levels of TNF-α and IL-1β in stool, and TNF-α in blood [5]. Thus, studies to date suggest that *V. parahaemolyticus* causes disease via different mechanisms than the related pathogen *V. cholerae*, which does not disrupt the intestinal epithelium nor induce significant inflammation in infected individuals (reviewed in [6]).

Comparison of clinical and environmental isolates of *V. parahaemolyticus* has enabled identification of several likely virulence factors in this pathogen. Of note, most clinical isolates exhibit β-hemolytic activity on Wagatsuma agar (*aka* the Kanagawa phenomenon) [7], [8], while this phenotype is lacking in most environmental isolates [9]. Hemolytic activity is mediated by the thermostable direct hemolysin (TDH) [10], and has also been demonstrated for the Tdh-related hemolysin (TRH) using human erythrocytes [11]. Recently, genome sequencing has revealed that pathogenic isolates of *V. parahaemolyticus* also encode two type III secretion systems (T3SS) [12]. T3SS have been found to contribute to the virulence of many bacterial pathogens, as they enable injection of bacterial proteins (effectors) directly into host cells and subsequent modulation of numerous host processes [13]. T3SS2 of *V. parahaemolyticus* is encoded in a pathogenicity island on the small chromosome (chrII) of pathogenic strains. This pathogenicity island also encodes TDH; consequently, the Kanagawa phenomenon is indirectly a marker for 2 distinct potential virulence factors. T3SS1 is encoded on the bacterium's large chromosome (chrI), and unlike T3SS2, appears to be ubiquitous within both clinical and environmental isolates of *V. parahaemolyticus* [12].

Several *in vitro* and *in vivo* assays have been used to characterize the effects of *V. parahaemolyticus* and its putative virulence loci upon host cells and tissues. *V. parahaemolyticus* causes a variety of changes in cultured cell lines, including release of cellular contents, cell rounding, disruption of tight junction complexes and cytoskeletal structures, and ion influx (reviewed in [14]). In particular, tissue-culture-based assays have been useful for linking T3SS1 with cytotoxicity [15]–[21], although the relationship between cytotoxicity and enteritis remains unclear. The effects of *V. parahaemolyticus* upon animal tissues have also been studied, primarily using rabbit ligated ileal loops, a model of intestinal fluid response. In this model, both TDH and T3SS2 contribute to fluid accumulation, with T3SS2 exerting the predominant effect [19], [22]. Studies using ligated ileal loops also suggest that T3SS2 is linked to inflammation and epithelial denudation. T3SS2 was likewise required to induce mild transient (∼4 hr) diarrhea in 2-day-old piglets orally infected with *V. parahaemolyticus*; however, pig intestines showed minimal histopathologic abnormalities, even when infected with wild type bacteria [23]. Limitations in each of these models indicate that studies of the pathogenesis of *V. parahaemolyticus*-linked enteritis would be transformed by development of a non-surgical small animal model in which bacterial colonization and host response, including diarrhea, histopathology and inflammation, could all be monitored and evaluated.

Here, we report that infant rabbits oro-gastrically inoculated with *V. parahaemolyticus* develop severe diarrhea and enteritis. The pathogen primarily colonizes the distal small intestine, the site where inflammation and dramatic histopathologic and ultrastructural changes in the epithelium were also observed. Similar to attaching and effacing (A/E) pathogens such as enteropathogenic *E. coli* (EPEC), which also cause intestinal disease, and in marked contrast to *V. cholerae, V. parahaemolyticus* causes effacement of the microvilli. However, *V. parahaemolyticus* causes more widespread disruption of villus structure than A/E pathogens. *V. parahaemolyticus* proliferates in epithelial cavities, initially forming large, dense microcolonies and ultimately inducing extensive extrusion and/or erosion of villous epithelial cells and a loss of epithelial barrier function in the small intestine. *V. parahaemolyticus* proliferates in cavities created in the epithelium forming large, dense microcolonies. T3SS2 proved to be essential for *V. parahaemolyticus* to colonize the small intestine and cause disease, but T3SS1 and TDH also modulate *V. parahaemolyticus* virulence in the intestine. When taken together, our findings suggest that *V. parahaemolyticus* damages the host intestine via a previously undescribed process and that infant rabbits are an outstanding model host for investigating *V. parahaemolyticus* pathogenicity.

## Results

### Oro-gastric infection of infant rabbits with *V. parahaemolyticus* causes severe diarrhea and enteritis

**Figure 1:**
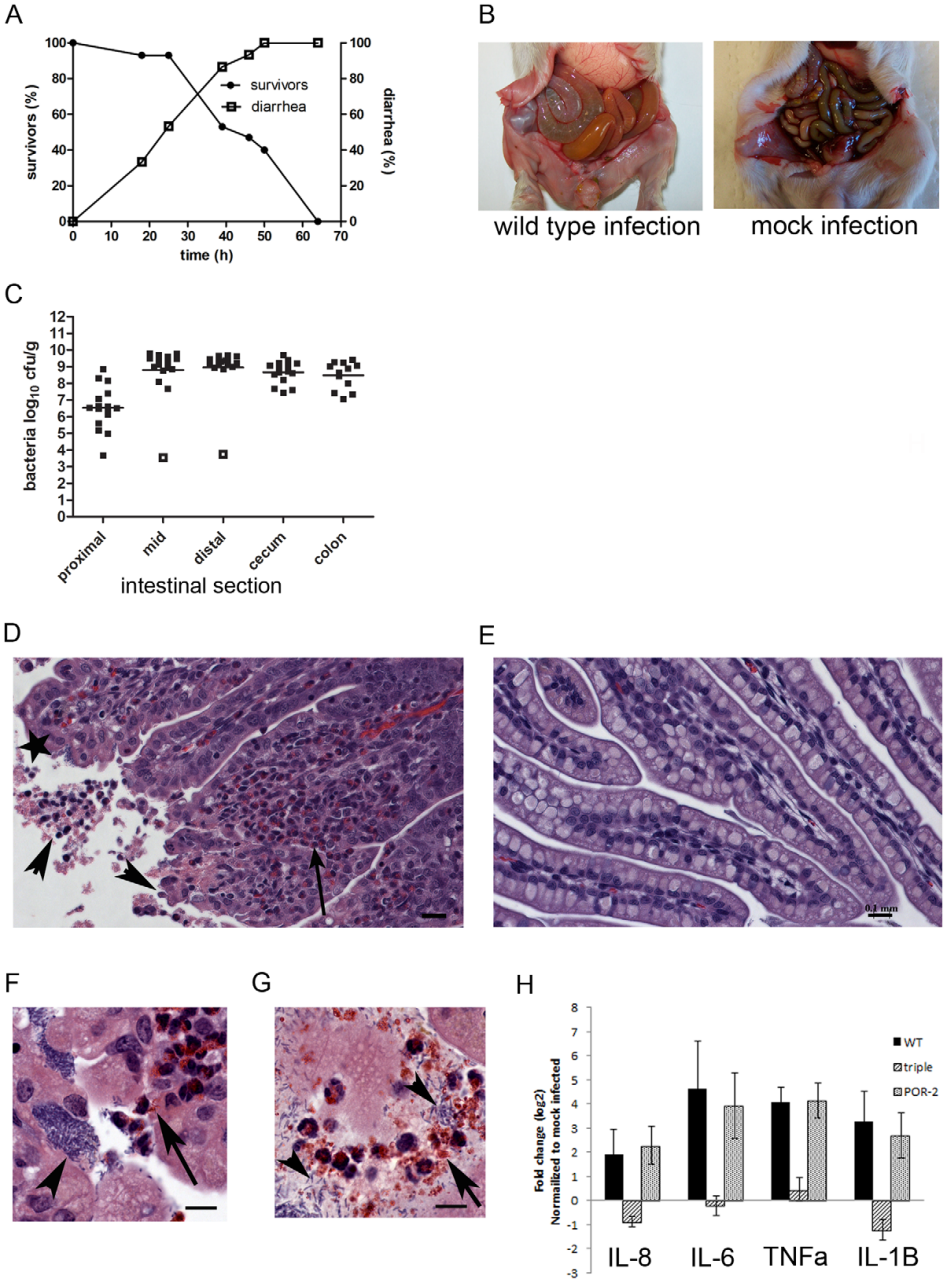
*V. parahaemolyticus* colonizes the small intestine of infant rabbits and induces a destructive enteritis. (A) Diarrhea and mortality in *V. parahaemolyticus*-infected rabbits over time (n = 15 rabbits). (B) Representative images of the intact intestine of *V. parahaemolyticus*-infected and mock-infected rabbits. (C) Recovery of *V. parahaemolyticus* (cfu g^−1^) from tissue homogenates from indicated intestinal section. Points represent individual rabbits; bars represent geometric means. Open boxes represent the limit of detection for samples from which no cfu were detected. (D–G) H&E-stained sections from the distal small intestine of infected (D, F–G) and mock infected (E) rabbits. In (D), the long arrow points to heterophils, arrowheads indicate sloughed or detaching host cells and the star indicates attached bacteria. In (F), the arrowhead indicates a cluster of bacteria and the arrow indicates heterophils traversing the epithelial barrier, whereas in (G), the arrowheads indicate bacteria and the arrow indicates degranulated heterophils. Scale bars are 100 µm and 10 µm in D–E and F–G, respectively. (H) Relative levels (mean ± average deviation) of transcripts for cytokines in homogenates of the distal small intestine from wild type (WT), the triple mutant (Δ*tdh* Δ*vcrD1* Δ*vcrD2*) and POR-2 (Δ*tdh* Δ*vscN1*) -infected rabbits. In B-H samples were collected at 38 hr PI.

We tested whether rabbits could be used as a model host to study *V. parahaemolyticus*-induced gastroenteritis, as we previously found that the intestinal diseases caused by *V. cholerae* O1, *V. cholerae* non-O1 non-O139, and enterohemorrhagic *E. coli* (EHEC) can be successfully modeled using these animals [24]–[27]. Based on our previous experience, 3-day-old rabbits were treated with cimetidine, a histamine H_2_ receptor antagonist that transiently alleviates gastric acidity, prior to bacterial inoculation [28]. Most rabbits oro-gastrically inoculated with *V. parahaemolyticus* developed severe diarrhea, but the clinical course and kinetics of disease in *V. parahaemolyticus*-infected rabbits differed from the diseases caused by *V. cholerae* O1, *V. cholerae* non-O1 non-O139 serogroups, or EHEC. In almost all rabbits, inoculation with 1×10^9^ colony forming units (cfu) of *V. parahaemolyticus* resulted in release of loose, unformed gelatinous stools, followed by liquid yellow diarrheal fluid, which typically developed between 20–40 hr after infection (Figure 1A) and soaked the ventral surfaces of the rabbits. If the experiments were continued, the animals subsequently lost weight, became lethargic and died by 64 hr (Figure 1A and Table 1). Thus, in most experiments described below, we used end points at or prior to 38 hr to enable a variety of analyses at times when most rabbits exhibited disease but had not yet succumbed to infection. At 38 hr post-infection (PI), the intestines of most surviving rabbits were swollen and filled with fluid (Figure 1B), with elevated fluid accumulation ratios versus mock-infected rabbits (Table 1). Notably, the fluid contained higher levels of total protein (0.53 vs 0.2 g dL^−1^) and K^+^ (35 vs 18 mmol dL^−1^) than the fluid from rabbits infected with *V. cholerae* [24]. The elevations in the levels of protein and K^+^ are consistent with the marked disruption of small intestinal villous epithelial cells observed in *V. parahaemolyticus*-infected animals (see below) and suggestive of a diarrheal mechanism that is distinct from the classic secretory diarrhea of cholera, where the intestinal epithelium remains intact [29].

**Table 1:** Diarrhea in rabbits following oro-gastric inoculation of *V. parahaemolyticus* or one of its isogenic derivatives.

Strain^a^	WT	mock	TDH	T3SS1	T3SS2	triple
Diarrhea (%)^b^	70	0	92	27^c^	0	0
Disease category^d^						
Dead	4	0	2	0	0	0
Diarrhea	10	0	9	4	0	0
Intestinal fluid	2	0	1	8	0	0
No gross disease	4	6	0	3	12	10
Total no. animals	20	6	12	15	12	10
FAR^e^	0.29±0.13	0.04±0.02	0.42±0.09	0.17±0.11	0.08±0.03	0.05±0.02
P-value: WT	-	P≤0.001	P≤0.05	P≤0.05	P≤0.001	P≤0.001
Mock	P≤0.001	-	P≤0.001	P≤0.05	NS	NS

a All strains are derivatives of *V. parahaemolyticus* RMID2210633 (WT); mutants contain the following gene deletions - TDH (Δ*tdhAS*), T3SS1 (Δ*vscN1*), T3SS2 (Δ*vscN2*) and triple (Δ*tdhAS* Δ*vcrD1* Δ*vcrD2*).

b Percentage of rabbits by 38 hr PI that had yellow, watery diarrhea.

c The proportion of rabbits with diarrhea was significantly (P<0.05; Fishers exact test) lower when infected with the T3SS1 mutant compared to the wild type.

d Number of rabbits within each disease category as described in the methods.

e Fluid accumulation ratio (FAR) of all infected (living) rabbits within the group (mean ± SD). FARs of wild type or mock infected rabbits were compared to mutants using one way ANOVA with Bonferroni's multiple comparison test.

The lower half of the small intestine appears to be the primary target of *V. parahaemolyticus*. The highest numbers of *V. parahaemolyticus* were recovered from homogenates of the mid and distal regions of the small intestine (∼10^9^ cfu g^−1^); ∼10–200-fold fewer cfu were recovered from the cecum, colon and proximal small intestine (Figure 1C). Additionally, histologic analyses revealed the more extensive pathologic changes to the tissue occurred within the lower third of the small intestine (Figure 1D). In hematoxylin and eosin (H&E)-stained sections from the distal small intestine, the tips of the villi appeared ragged and irregular, and epithelial cell debris from the disrupted villi was detected in the intestinal lumen (compare Figures 1D & E). Discrete and oftentimes dense clusters of bacteria adherent to the epithelium were also evident in these sections (Figure 1F, arrowhead). Heterophils, the rabbit equivalent of neutrophils, were observed in the lamina propria as well as apparently migrating through the epithelial barrier toward adherent bacteria and the intestinal lumen (Figure 1F arrow); degranulated heterophils were seen as well (Figure 1G). Consistent with the histologic evidence of marked inflammation, 4–20 fold elevations in the abundance of transcripts for IL-8, IL-6, TNF-α, and IL-1β by quantitative PCR analyses of RNA were found in homogenates of the distal small intestines of infected relative to uninfected rabbits (Figure 1H). In addition, the zone of proliferating cells at the base of the villi was larger in infected animals than in controls, suggesting that *V. parahaemolyticus* infection induces host cell proliferation (see below). Pathological abnormalities in the cecum were limited to moderate sub-mucosal edema which contained few inflammatory cells. Importantly, attached bacteria and histologic abnormalities were not observed in the colon, despite large numbers of bacteria recovered from tissue homogenates taken from this site. Finally, blood samples and homogenates of the spleen, gall bladder and liver rarely contained *V. parahaemolyticus* (bacteria were recovered in 3 of 16 samples at approx. 10–100 cfu mL^−1^ homogenate), suggesting that the infection does not ordinarily extend beyond the intestine in the infant rabbits. Collectively, these observations show that *V. parahaemolyticus* adheres to and colonizes the distal small intestine of the infant rabbit, where it induces inflammatory enteritis accompanied by severe disruption of the epithelial lining.

### Kinetics of *V. parahaemolyticus*-induced intestinal pathology and colonization

**Figure 2:**
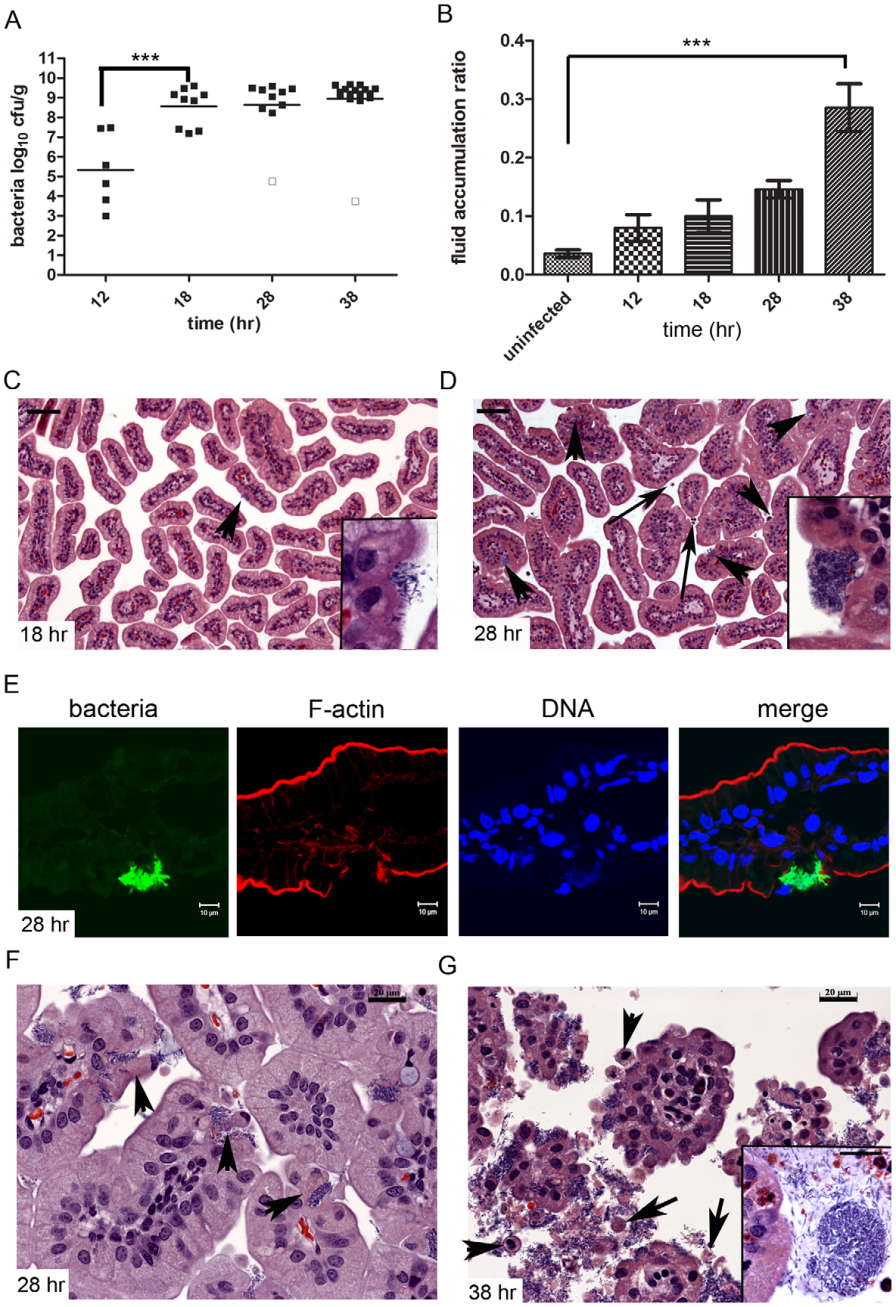
Kinetics of fluid accumulation and pathology in *V. parahaemolyticus*-infected infant rabbits. (A) Concentration (cfu g^−1^) of *V. parahaemolyticus* recovered in distal small intestine homogenates at different times after inoculation. Data points represent individual rabbits; bars represent geometric means. Open boxes represent the limit of detection for samples from which no cfu were isolated. Groups were compared using one-way ANOVA with Boneffoni's multiple comparison post-test (*** P<0.001). (B) Fluid accumulation ratio (mean ± SEM) in the distal small intestine of rabbits at various times PI. Data were compared to uninfected rabbits using one-way ANOVA with Boneffoni's multiple comparison post-test (*** P<0.001). (C–D and F–G) Representative H&E-stained sections from the distal small intestine of infected rabbits at 18 hr (C), 28 hr (D, F) and 38 hr PI (G) showing the frequency and size of attached bacterial clusters as well as histological changes. Arrowheads in C and D point to bacterial microcolonies and long arrows indicate luminal heterophils; in F, arrowheads point to extruding epithelial cells with *V. parahaemolyticus* at their base, and in G, arrowheads point to extruding cells and long arrows point to cytoplasmic fragments. Scale bars are 100 µm and 20 µm in C–D and F–G, respectively. (E) Small intestinal tissue sections from rabbits infected with *V. parahaemolyticus* expressing GFP (green) counterstained with phalloidin (red) and DAPI (blue) to detect F-actin and nuclei acid, respectively.

To gain insight into the temporal progression of *V. parahaemolyticus*-linked pathological changes in the small intestine, as well as their relationship to overt signs of disease, we compared tissue from the distal small intestines of infected rabbits at 12, 18, 28 and 38 hr PI. Bacterial numbers within tissue homogenates increased markedly between 12 and 18 hr PI (Figure 2A.) After 18 hr PI, the number of organisms recovered from the distal small intestine remained relatively constant, despite the onset of diarrhea (and the loss of bacteria-laden fluid containing 10^9^ cfu mL^−1^) at ∼28 hr; thus, bacterial proliferation clearly continues despite the absence of an increase in the measured bacterial load within the intestine. The amount of fluid accumulated in the distal small intestine increased gradually, reaching statistically greater levels than in mock-infected rabbits at 38 hr PI (Figure 2B).

Bacterial abundance as assessed by plating tissue homogenates only partially corresponded to the microcolonies of bacteria evident in H&E-stained tissue sections. Bacterial microcolonies were not usually detected in tissue sections at 12 hr PI; however, by ∼18 hr PI, small clusters were more frequently found on the epithelial surface (Figure 2C). By 28 hr PI, the bacterial microcolonies were larger and more abundant, and appeared to be located within ‘cavities’ on the epithelial surface (Figure 2D–F). The observed increase in visible bacterial microcolonies at 28 hr relative to 18 hr, which did not correspond with any further increase in the concentration of bacteria recovered in tissue homogenates, may reflect better attachment of the bacteria by 28 hr or better retention of bacteria in the cavities during staining of tissue sections. Finally, at 38 hr PI, large numbers of bacteria, both as individual cells and as large (sometimes exceeding 50 µm in diameter), dense microcolonies, were associated with the villi and the luminal cellular debris (Figure 2G). Bacteria within tissue sections were confirmed to be *V. parahaemolyticus* via infection of rabbits with a strain that constitutively expresses green fluorescent protein (GFP); confocal microscopy analyses revealed that the distribution of GFP-expressing bacteria, both within cavities on the epithelial surface (Figure 2E), and in association with epithelial debris corresponded closely to that seen in H&E-stained sections. Furthermore, no bacteria were seen in mock-infected rabbits at any point.

Serial histologic analyses also revealed a striking characteristic progressive disruption of the small intestine's epithelial morphology during the infection. At 12 and 18 hr PI, intestinal villi appeared intact, with a fairly smooth surface, although by 18 hr, heterophils were observed in the lamina propria of villi, where small clusters of bacteria were also often observed to be attached (Figure 2C, data not shown). Even at this earlier time point, adherent bacterial clusters were associated with erosions in the epithelial surface (Figure 2C inset). By 28 hr PI, these erosions were more pronounced, generating cavities in the epithelial surface and resulting in loss of the peripheral actin ring that ordinarily encircles each villus (Figure 2D (inset), 2E). Formation of these cavities appears to rely on extrusion of epithelial cells and/or their contents (Figure 2F). *V. parahaemolyticus* cells were often observed at the base of extruding epithelial cells (Figure 2F, arrowheads). At 28 hr, heterophils had become more abundant within the lamina propria, and a few were observed in the intestinal lumen (Figure 2D, long arrows). However at this point, most of the epithelial layer remained intact and little luminal debris was observed.

Extensive disruption of the epithelium and disintegration of villi was not evident until 38 hr PI; at this point, bacteria were often associated with epithelial cells that were loosely connected together, giving villi a ‘flower-like’ appearance, and with the abundant epithelial debris within the intestinal lumen (Figure 2G, also Figure 1D). Luminal debris consisted of both whole cells (with nuclei) and membrane-bound fragments of cytoplasmic material (Figure 2G). At this time point, while increasing numbers of heterophils continued to be recruited into the intestine, it was somewhat surprising that more were not present given the amount of tissue destruction that was observed. However, immunostaining revealed that macrophages, which were present in low numbers at earlier time points, were more abundant at 38 hr PI, both in the tissue and in the intestinal lumen (Figure S1). Collectively, these analyses suggest that the initial attachment of *V. parahaemolyticus* to discrete areas in the distal small intestine initiates a cascade of changes in the host. As the pathogen adheres and proliferates, small surface erosions give rise to bacterial-filled cavities in the epithelium, followed by disintegration of the villi. An acute (heterophil-based) inflammatory response occurs at the same time as early epithelial surface erosion, when a relatively small number of bacteria are attached to the surface. Thus, the inflammatory response to *V. parahaemolyticus* infection precedes, rather than occurs as a result of, the extensive tissue disruption observed at later (e.g., 38 hr) stages in the infection.

In normal tissue, the loss of dying or damaged epithelial cells from the villus is balanced by the generation of new progenitor cells by proliferation in the crypts. Thus, to explore the relationship between intestinal cell proliferation and epithelial cell loss, we also assessed cell proliferation at 18 and 28 hr PI. Tissue sections were labeled with anti-Ki67, an intrinsic marker for actively dividing cells [30], and the zone of proliferating cells relative to villus length was calculated (Figure S2). Strikingly, even at 18 h PI, the zone of proliferating cells was significantly greater in infected compared to uninfected rabbits (mean ± SEM: 0.52±0.06 and 0.21±0.02; P<0.01). This difference was also apparent at 28 hr PI (0.65±0.04 and 0.36±0.06; P<0.05). (We did not perform Ki67 staining at 38 hr PI due to the massive tissue disruption that had occurred). These data strongly suggest that epithelial cell proliferation in response to *V. parahaemolyticus*, like the inflammatory response, does not occur as a result of tissue disruption, but is instead an early step in the host response to infection.

### *V. parahaemolyticus* induces striking ultrastructural changes in villus epithelial cells

**Figure 3:**
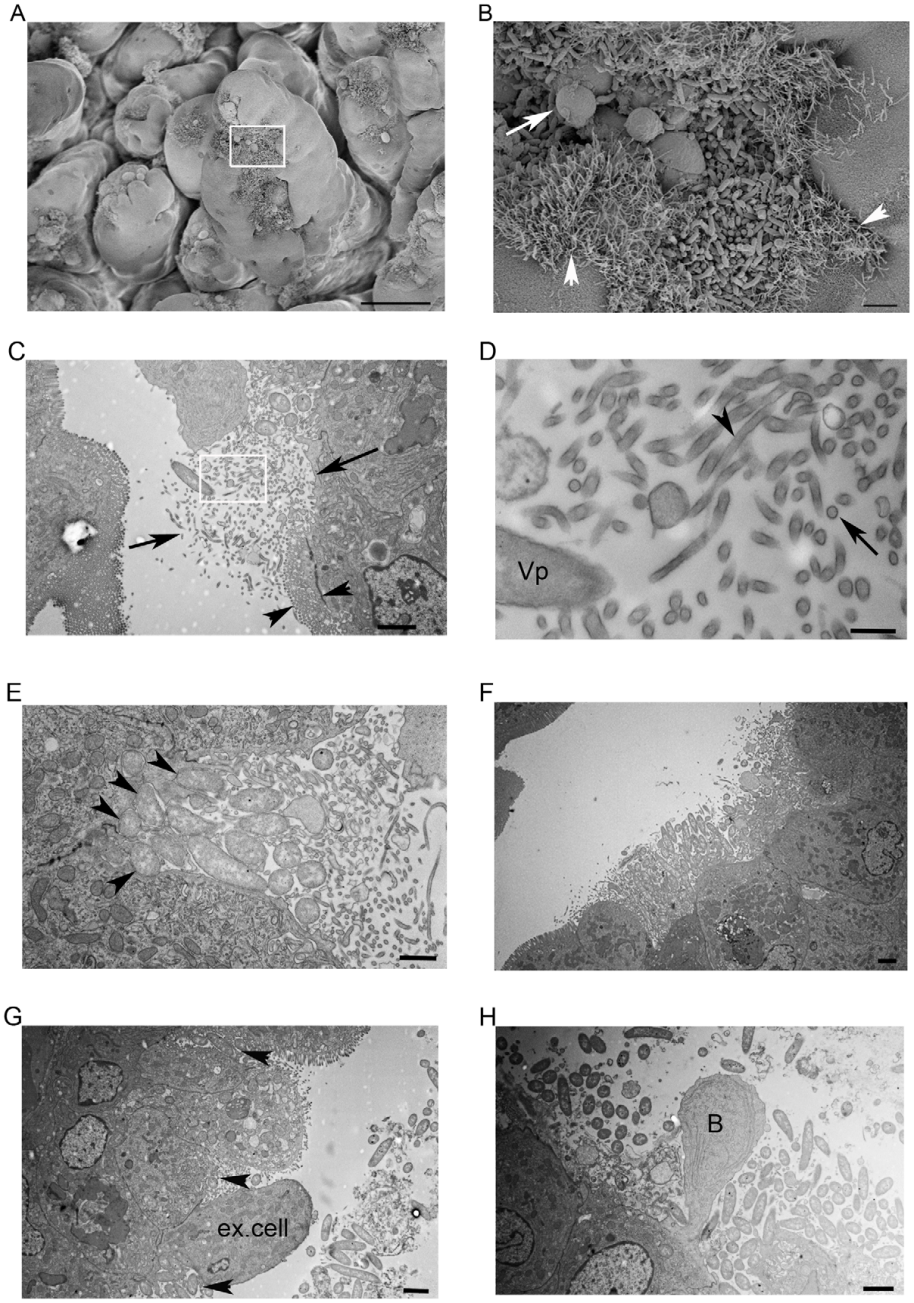
Representative electron micrographs of *V. parahaemolyticus* attached to the intestinal epithelium at 28 hr PI. (A) Scanning electron micrograph of the small intestine of *V. parahaemolyticus*-infected infant rabbits. Scale bar = 50 µm. (B) Higher magnification image of the boxed area in (A) showing elongated microvilli (arrowheads) and ‘blebs’ of material being lost from the epithelial surface (long arrow). Scale bar = 2 µm. (C) Transmission electron micrograph of *V. parahaemolyticus* colonizing the epithelial surface, surrounded by a tangle of elongated microvilli-like projections. Note the organized pattern of microvilli (seen in cross-section) on adjacent uninfected cells (arrowheads) compared to the disorganized mix of microvilli sections, cytoplasmic debris and bacteria that extends much further into the luminal space (long arrows). Scale bar = 2 µm. (D) Higher magnification image of the boxed area in (C) showing the defined membranes and presence of internal filaments (dark staining) in cross-sections (long arrow) and longitudinal sections (arrowhead) of elongated microvilli. Vp = bacterium. Scale bar = 100 nm. (E) Close contact between bacteria and host epithelial cell membranes (arrowheads) in the absence of normal microvilli and pedestal formation. Scale bar = 1 µm. (F) Clusters of *V. parahaemolyticus* located in a cavity below the normal level of the surrounding epithelium. Note the intact brush border of adjacent cells. Scale bar = 2 µm. (G) *V. parahaemolyticus* (arrowheads) located at the base of an extruding epithelial cell (ex. cell), with more bacteria in the intestinal lumen. Scale bar = 2 µm. (H) Membrane-bound cytoplasm ‘bleb’ (B) extruding from the epithelium, surrounded by *V. parahaemolyticus*. Scale bar = 2 µm.

To further characterize how *V. parahaemolyticus* interacts with epithelial cells, we used scanning and transmission electron microscopy (EM) to visualize the epithelium in the small intestine of infected rabbits. Numerous clusters of attached bacteria were observed with scanning EM by 28 hr PI, particularly near the villus tips (Figure 3A); bacteria were not observed within the crypts. Epithelial cells with attached *V. parahaemolyticus* exhibited dramatic ultrastructural changes, whereas epithelial cells without bacteria appeared normal (Figure 3B, data not shown). Attached bacteria were typically found in clusters, with individual cells often oriented perpendicular to the epithelial surface (Figure 3B). Notably, the appearance of the epithelial surface surrounding bacterial clusters was grossly distorted by the presence of numerous elongated (5–10 µm long) hair-like cellular projections (Figure 3B, arrowheads; Figure S3A). In transmission EM, depending on the angle of sectioning, these protrusions appear as a disorganized mix of cross-sections or short fragments that extend well beyond the range of the normal brush border (Figure 3C). Higher magnification images revealed that these structures were surrounded by a clearly defined membrane and contained internal filaments (Figure 3D), two features that strongly suggest that these protrusions represent elongated microvilli. Similar but shorter projections, also thought to represent elongated microvilli, were observed around clusters of EPEC attached to human duodenal tissue explants [31], [32]. Although *V. parahaemolyticus* induces elongation of microvilli from the surface of epithelial cells near attached bacteria, in virtually all EM images, *V. parahaemolyticus* was only closely apposed to host cells without detectable microvilli (Figure 3E). Microvilli loss (effacement) appears to occur by the process of membrane vesiculation [33], as vesicles with poorly defined membranes and lacking core proteins were observed near the attached bacteria (Figure S3B).

Some *V. parahaemolyticus* cells closely apposed to the epithelial cell membrane appeared to be held in ‘cup-like’ structures that are reminiscent of the lesions caused by A/E pathogens (Figure 3E, arrowheads). However, with *V. parahaemolyticus* there was limited evidence of actin accumulation beneath the attached bacteria and pedestals were never observed, suggesting that the adherence mechanisms of these two enteric pathogens are not equivalent. In addition, the villi destruction caused by *V. parahaemolyticus* (which contrasts dramatically to the intact epithelium of tissue infected with *V. cholerae* O1 ([34]; Figure S3C)) is far more severe and widespread than is typically seen with A/E pathogens. Consistent with our observations with H&E-stained sections, in transmission EM images, bacterial clusters were typically seen beneath the level of the surrounding (intact) epithelium (Figure 3F). EM evidence for both extrusion of epithelial cells (Figure 3G) and their contents (see Figures 3B (arrow) and 3H) was observed, suggesting that both processes contribute to the development of cavities in the epithelial surface.

### *V. parahaemolyticus* induces re-distribution of cytoskeletal and tight junction proteins

**Figure 4:**
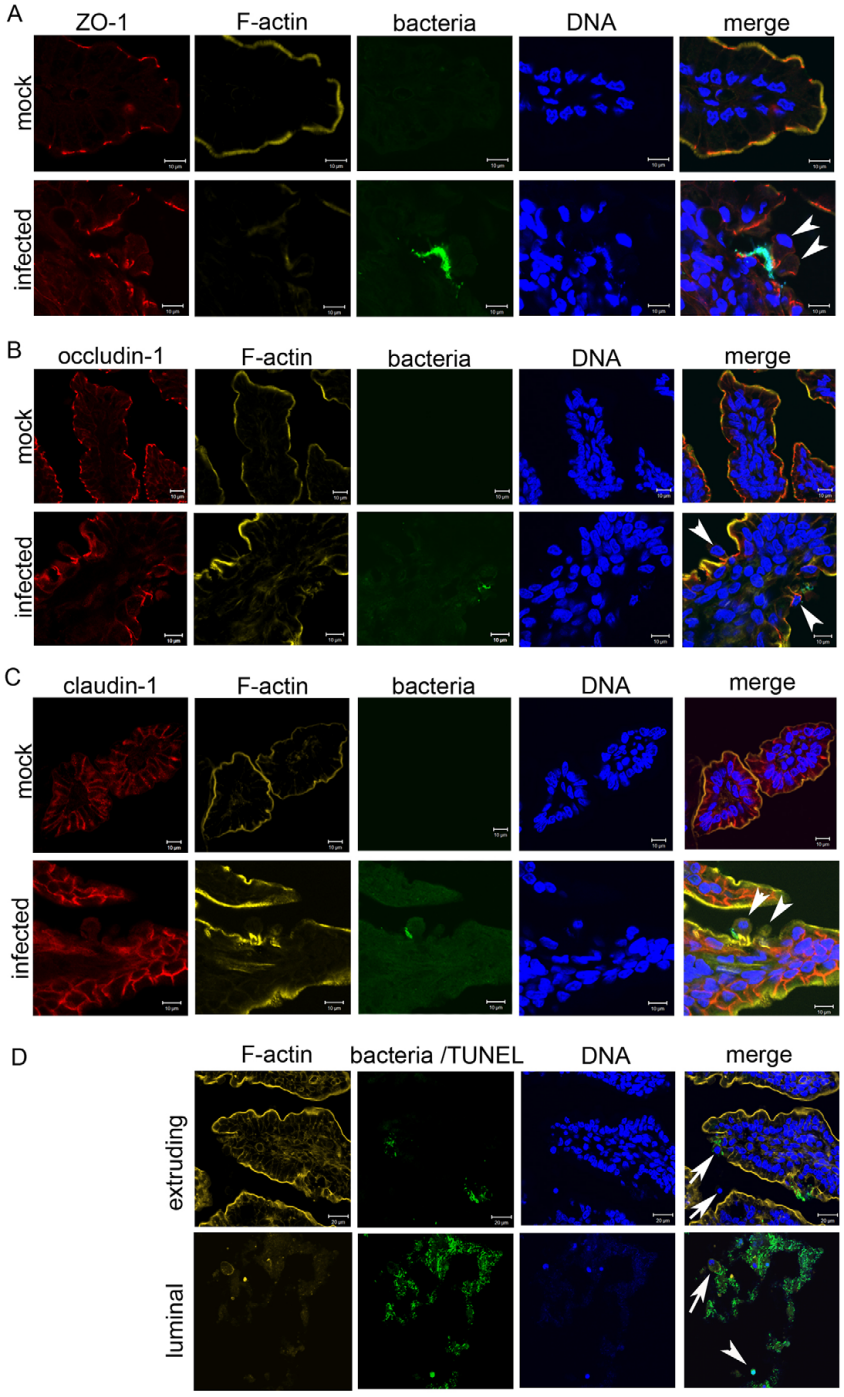
Extruding epithelial cells show redistribution of some tight junction proteins but lack markers of apoptosis. (A–C) Immunofluorescence micrographs of small intestinal sections from mock and infected rabbits stained for (A) ZO-1, (B) occludin-1 and (C) claudin-1. Note altered localization of ZO-1 and occludin-1 but not claudin-1 in extruding epithelial cells with adjacent *V. parahaemolyticus* (arrowheads). Actin cytoskeleton was stained with phalloidin-Alexa 568 (yellow), bacteria constitutively expressed green fluorescent protein (green) and nuclei were stained with DAPI (blue). (D) Tissue sections from infected rabbits were stained for apoptotic cells using the TUNEL assay. TUNEL-positive cells were detected using a fluorescein-based detection system thus both bacteria and apoptotic host cells appear green, but can be distinguished based on their relative size and shape. Examples of TUNEL-positive (arrowhead) and TUNEL-negative (arrows) host cells are indicated. Sections were counterstained with phalloidin-Alexa 568 (yellow) and DAPI (blue) as described above.

Cell shedding is a normal process in which apoptotic epithelial cells are routinely extruded into the lumen [35]. Re-distribution of cytoskeletal proteins (e.g., actin) as well as proteins that form apically located tight junction complexes (e.g., ZO-1, claudins and occludin-1) is thought to contribute to cell shedding while maintaining the integrity of the epithelial barrier [36], [37]. Therefore, to begin to investigate the molecular processes that underlie *V. parahaemolyticus*-induced cell extrusion from the small bowel epithelium, we compared the localization of host cytoskeletal components in tissues from infected and mock-infected rabbits. Re-distribution of F-actin, ZO-1, and occludin-1 from their normal locations at the apical cell periphery and the apical boundaries between cells to the lateral membrane was apparent for cells undergoing extrusion. The redistributed proteins formed an intense focal point or funnel-like structure at the base of the extruding cell (Figure 4A, B), similar to structures that have been observed during both physiologic and pathologic epithelial cell shedding [36], [37]. These observations suggest that *V. parahaemolyticus* may usurp at least certain components of the normal shedding process to promote cell extrusion during the course of infection. However, in contrast to ZO-1 and occludin-1, claudin-1, which is located along the lateral membranes in uninfected tissue, did not appear to co-localize with F-actin at the lateral membrane as occurs in physiologic and pathologic cell shedding (Figure 4C; [37]). The claudin family of proteins is thought to control the paracellular permeability of tight junctions [38], [39] and so the aberrant localization of claudin-1 in extruding cells of infected tissues may contribute to fluid loss. Furthermore, while shedding cells normally exhibit signs of apoptosis [35], [40], most cells undergoing *V. parahaemolyticus*-induced extrusion did not, as no TUNEL staining was detected in extruding cells (Figure 4D). Thus, *V. parahaemolyticus* appears to elicit extrusion of cells that would not ordinarily undergo shedding. TUNEL-positive cells were present in the luminal debris (Figure 4D) especially at later points in the infection, suggesting that cell death occurs after extrusion during *V. parahaemolyticus* infection.

### *V. parahaemolyticus* disrupts epithelial barrier integrity in the small intestine

**Figure 5:**
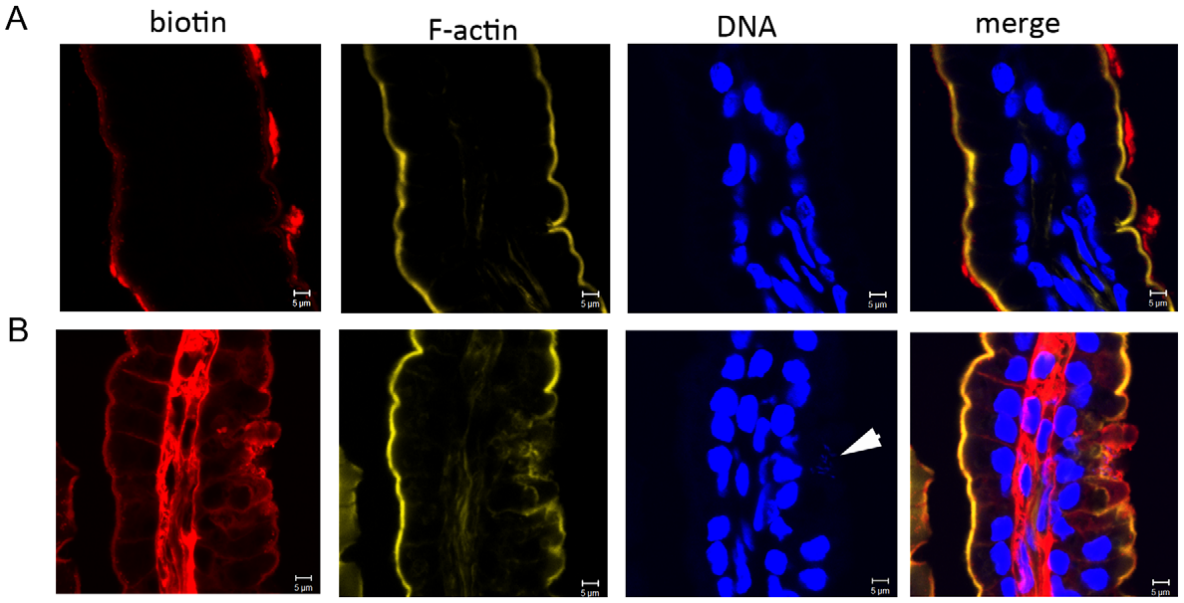
*V. parahaemolyticus* compromises epithelial barrier function in the small intestine. Penetration of biotin (red) into the lamina propria in mock-infected (A) or *V. parahaemolyticus*-infected rabbits (B) at 25 hr PI. Tissues were counterstained with DAPI (blue) and phalloidin-Alexa 568 (yellow) to stain nucleic acid and F-actin, respectively. Note that biotin is located within the villi in sections from infected rabbits, but not in control rabbits. Adherent bacteria are marked with an arrowhead.

Our observations of increased epithelial cell shedding and the formation of bacteria-filled cavities in the epithelium of *V. parahaemolyticus*-infected rabbits raised the possibility that infection might compromise epithelial barrier function, even when the intestinal tissue appears largely intact. Consequently, we tested whether a small molecular tracer, biotin, could penetrate beyond the luminal surface of the epithelium after injection into the dissected distal small intestine or colon of infected and control rabbits at 25 hr PI. In the mock-infected rabbits, biotin remained localized to the luminal side of the epithelial barrier in the small intestine and colon (Figure 5A; data not shown). In contrast, biotin was detected in the lamina propria as well as on the luminal surface of tissue from the small intestine of *V. parahaemolyticus*-infected rabbits (Figure 5B), even though at the time point assayed the villi structure was not extensively disrupted. The route by which biotin reaches the lamina propria cannot definitively be discerned from this experiment; however, *V. parahaemolyticus*-induced disruptions in the proteins that form the tight junction complex, and a resulting increase in paracellular permeability, seem likely to play an important role. No penetration of biotin was observed in the colon of infected rabbits, consistent with the previously noted lack of bacterial attachment and pathology at this site (Figure S4). This finding provides additional evidence that the small intestine is the pathologically relevant site during *V. parahaemolyticus* infection.

### *V. parahaemolyticus* T3SS2 is required for colonization and enteritis

**Figure 6:**
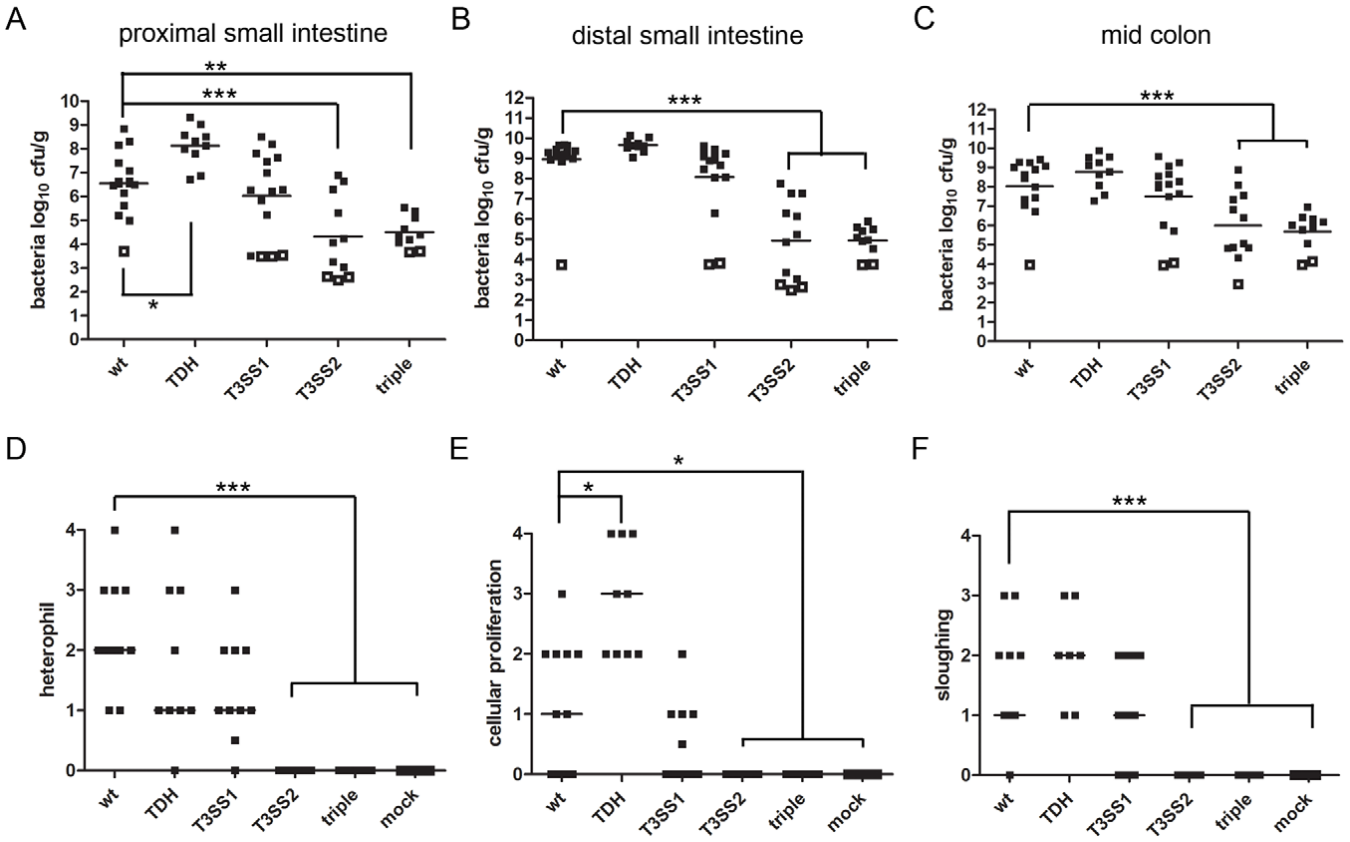
Colonization and pathological changes induced by wild type *V. parahaemolyticus* and isogenic mutants at 38 hr PI. (A–C) Concentration (cfu g^−1^) of bacteria recovered from tissue homogenates of intestinal samples from rabbits infected with wild type *V. parahaemolyticus* or from mutants lacking one or more putative virulence factors. Data points represent individual rabbits. Open symbols represent the limit of detection for samples from which no cfu were isolated. Bars show the geometric mean. Statistical analysis was performed using one way ANOVA and Bonferroni's multiple comparison post-test. *P≤0.05, **P≤0.01 and ***P≤0.001. (D–F) Pathologic scores for abnormalities in the small intestine of infected or mock-infected rabbits. Data points represent individual rabbits. Statistical analysis was performed using Kruskal-Wallis statistic with Dunn's post-test for multiple. *P≤0.05, **P≤0.01 and ***P≤0.001.

To begin to address which bacterial factors are important in *V. parahaemolyticus*-induced enteritis, we assessed colonization by and disease associated with a previously described set of isogenic *V. parahaemolyticus* mutants, each lacking one or more of the 3 principal described virulence factors: TDH (Δ*tdh*), T3SS1 (Δ*vscN*1, an essential component of T3SS1) and T3SS2 (Δ*vscN*2, an essential component of T3SS2) [22]. A triple Δ*tdh* Δ*vcrD*1 Δ*vcrD*2 mutant (*vcrD*1 and *vcrD*2 are also essential components of T3SS1 and T3SS2, respectively) did not cause disease or elicit inflammation in rabbits (Table 1, Figure 6). Of the single deletions, inactivation of T3SS2 had the most dramatic attenuating effect, which closely resembled that of the triple mutation. Of 12 rabbits infected with the Δ*vscN*2 mutant, none developed diarrhea, exhibited intestinal fluid accumulation, or induced histological changes in intestinal tissue (Table 1, Figure 6 and Figure S5). Notably, there was a more than 4-log reduction in the number of Δ*vscN*2 mutant bacteria recovered from the small intestine relative to the wild type strain (Figure 6A–B). Thus, the T3SS2 is essential for *V. parahaemolyticus* intestinal colonization. As such, it is likely that the profound attenuation of virulence of the Δ*vscN*2 mutant at least in part reflects its reduced capacity for intestinal colonization. However, since previous studies showed that T3SS2 accounted for most of the epithelial disruption and inflammation in ileal loops [19], a closed system in which many colonization factors may not be essential, it is likely that T3SS2 plays a critical role both in colonization and subsequent events in pathogenesis.

Inactivation of T3SS1 had a more subtle effect; however, our analyses suggest that this apparatus also contributes to *V. parahaemolyticus*-induced enteritis. Intestinal colonization by the Δ*vscN*1 mutant appeared to be slightly lower than that of the wild type strain, although this difference was not statistically significant (Figure 6A–C). As visualized by scanning EM, the Δ*vscN1* mutant induced microvillous elongation and formed microcolonies that were grossly indistinguishable to those observed during wild type infection. However, there was a significant reduction in the percentage of rabbits with diarrhea (27% vs 70% for the wild type strain). Additionally, rabbit intestines contained significantly less fluid when infected with the Δ*vscN*1 mutant, as reflected by the fluid accumulation ratio within the distal small intestine, although unlike the case with the Δ*vscN*2 mutant, some fluid was still observed (Table 1). Inactivation of T3SS1 had no apparent effect on heterophil infiltration, epithelial cell sloughing, or cell proliferation (Figure 6D–F; Figure S5). Furthermore, a mutant lacking T3SS1 (and also TDH, known as POR-2) showed no reduction in cytokine production relative to the wild type (Figure 1G), suggesting that the secretion system is not required for *V. parahaemolyticus* to induce an inflammatory response.

In contrast to T3SS2 and T3SS1, our data suggests that TDH dampens at least some aspects of the host response to *V. parahaemolyticus* infection. Rabbits infected with *V. parahaemolyticus* lacking TDH developed diarrhea and had a fluid accumulation ratio significantly greater than that of wild type bacteria (Table 1). Compared to the wild type, similar numbers of the *tdh* mutant were recovered in most intestinal sections; however, significantly higher numbers were recovered in the proximal small intestine suggesting that this mutant is better able to colonize this region of the intestine (Figure 6A). By scanning EM, TDH does not appear to be necessary for microvillous elongation or the formation of microcolonies on the epithelial surface as these features were grossly similar in rabbits infected with the wild type or the *tdh* mutant. Heterophil recruitment and epithelial cell sloughing were also not altered in response to the *tdh* mutant relative to the wild type strain (Figure 6D, F; Figure S5); however, in places, the superficial mucosa appeared necrotic, suggesting a more severe pathological reaction was occurring (Figure S6). Additionally, the zone of proliferating cells was significantly enlarged in response to the *tdh* mutant (Figure 6E; Figure S5). The presence of augmented proliferation in the absence of increased heterophil infiltration observed with the *tdh* mutant suggests that these two responses to *V. parahaemolyticus* infection occur independently.

## Discussion

We have developed a simple non-surgical oral infection model of *V. parahaemolyticus*-induced intestinal pathology and diarrhea. This experimental model enabled us to define several previously unknown but key features of the pathogenesis of the disease elicited by this common agent of seafood-borne gastroenteritis. First, we discovered that this organism chiefly colonizes the distal small intestine, the region of the intestine that is also the major site of *V. parahaemolyticus*-induced damage, increased permeability of the epithelial barrier and inflammation. Together, these observations strongly suggest that disease in this region of the gastrointestinal tract accounts for most, if not all, of the diarrhea that accompanies *V. parahaemolyticus* infection. Second, we found that the *V. parahaemolyticus* T3SS2 is essential for the pathogen to colonize the small intestine; prior to our work no *V. parahaemolyticus* intestinal colonization factors had been definitively identified because of the absence of a robust animal model. Third, we observed that *V. parahaemolyticus* causes marked disruption of the villous epithelial surface in the small intestine. Effacement of microvilli, re-distribution of cytoskeletal and tight junction proteins, and extrusion of epithelial cells in the small intestine all appear to contribute to villus disruption and the breakdown of epithelial barrier function. Furthermore, the pathogen induces remarkable elongation of microvilli in epithelial cells adjacent to attached *V. parahaemolyticus*. Finally, early in the infection, before widespread damage to the epithelium becomes evident, *V. parahaemolyticus* induces both proliferation of intestinal epithelial cells and recruitment of inflammatory cells. Thus, our observations suggest that *V. parahaemolyticus* elicits disease via a previously undescribed sequence of events that, to our knowledge, differs from those outlined for other enteric pathogens.

*V. parahaemolyticus*, like *V. cholerae* O1 [24], preferentially colonizes the distal small intestine. However, the manner in which these two pathogenic vibrios associate with the host epithelial surface differs. Prototypical *V. cholerae* O1 colonizes as layers of cells embedded in mucin-rich material that covers much of the epithelial surface of the villi as well as the crypts [24]. In contrast, *V. parahaemolyticus* colonizes as more discrete clusters of bacteria (*i.e.* microcolonies) that predominantly localize to the upper half of the villi; furthermore, unlike *V. cholerae* O1 [24], *V. parahaemolyticus* does not induce goblet cell degranulation. The mechanism(s) that hold the *V. parahaemolyticus* microcolonies together are not known. However, since the microcolonies appear to form during contact with the epithelial surface, it is tempting to speculate that *V. parahaemolyticus* lateral flagella, which can be induced by surface contact [41], [42], may promote microcolony formation. These lateral cell appendages have been reported to form linkages between neighboring bacteria and the surface [43]. Induction of peritrichous flagella is associated with conversion of *V. parahaemolyticus* from small (2–3 µm), polarly flagellated swimmer cells to swarmer cells, which are elongated (5–20 µm) as well as peritrichously flagellated [42], [44]. Interestingly, we observed *V. parahaemolyticus* cells up to 10 µm in length in electron micrographs of infected tissues (Figure S3D, asterisk), consistent with the idea that these cells have switched from the swimmer to the swarmer cell state.

**Figure 7:**
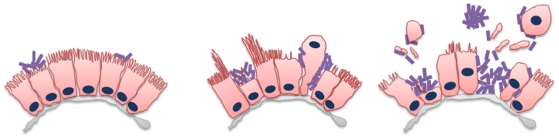
Schematic of kinetics of *V. parahaemolyticus*-induced damage to the intestinal epithelial surface. Following initial attachment, *V. parahaemolyticus* induces erosion of microvilli and depletion of cytoplasmic contents resulting in the formation of bacterial clusters located just below the level of the surrounding epithelium. Continued depletion of epithelial cell contents either by cytoplasmic ‘blebbing’, whole cell extrusion and microvilli elongation around the edge of the cluster, leaves *V. parahaemolyticus* clusters situated within deeper cavities in the epithelium. Eventually, this leads to disintegration of normal villus structure and the generation of large amounts of luminal debris. These pathological changes appear to be attributed to T3SS2 as a similar pathology was observed in rabbits infected with mutants lacking TDH or T3SS1. The purple rods represent *V. parahaemolyticus*.

The consequences of *V. cholerae* O1 and *V. parahaemolyticus* colonization of the small intestine are also fundamentally different. *V. cholerae* O1 does not damage the surface of the intestinal epithelium; instead, the cholera pathogen grows on the luminal side of the microvilli which remain largely intact ([34] and see Figure S3C). In marked contrast, *V. parahaemolyticus* damages the epithelial surface, leading to villus disintegration. Our histologic analyses of samples from 12 to 38 hr PI suggest that there is a characteristic sequence of steps through which *V. parahaemolyticus* proceeds to cause villus disruption in the small intestine (Figures 2 and 7). Initial attachment of *V. parahaemolyticus* to the epithelial surface is associated with effacement of microvilli and apparent depletion of cytoplasmic contents; thus, even at 18 hr PI, attached *V. parahaemolyticus* was observed situated below the epithelial surface (Figure 2C). Both marked depletion of epithelial cell cytoplasmic contents as well as epithelial cell extrusion contribute to the formation of these *V. parahaemolyticus*-filled cavities in the epithelial surface. It is not clear if *V. parahaemolyticus* penetration of the paracellular space to reach the base of the extruding cell is a required step for extrusion, though this was often observed (Figures 2F and 3G). The benefit of the epithelial cavities for the pathogen is not known, but it seems plausible that the cavities may provide the pathogen increased access to nutrients, or serve as a niche, offering protection from peristaltic flow.

The molecular mechanisms by which *V. parahaemolyticus* elicits epithelial cell extrusion are not known. The process bears some similarity to the normal process that leads to shedding of apoptotic epithelial cells [35]. *V. parahaemolyticus*-induced cell extrusion, as in physiologic cell shedding, is accompanied by redistribution of tight junction-associated proteins, including ZO-1 and occludin-1, towards the basolateral membrane where they co-localized with actin to form a funnel-like structure. However, claudin-1 did not follow this pattern in extruding cells of infected tissues, as occurs in physiologic and pathologic shedding [37]. Furthermore, while paracellular barrier integrity is maintained during physiological cell shedding, there is an increase in paracellular permeability observed in infected rabbits. *V. parahaemolyticus*-induced cell shedding could be a direct effect of the pathogen (e.g., perhaps a consequence of the activities of translocated T3SS effectors on tight junction complexes), or an indirect consequence of infection. For example, certain pro-inflammatory cytokines including TNF (whose production is stimulated by *V. parahaemolyticus*) have been shown to elicit cell shedding [37], [45]. Regardless of the mechanisms leading to epithelial cell extrusion, *V. parahaemolyticus*-induced break down in epithelial barrier function, which has also been observed in a polarized epithelial monolayer [46], likely contributes to the loss of intestinal fluid (diarrhea) caused by *V. parahaemolyticus*.

Pathogen-induced epithelial cell extrusion has also recently been detected for other enteric pathogens. For example, EHEC induces extrusion of cells from polarized monolayers and during infection of calves [47], [48]. Tissue-culture-based studies have revealed that the EHEC T3SS effector EspM, which interferes with the RhoA-signaling pathways that regulate actin cytoskeleton dynamics in eukaryotic cells, is sufficient to cause extrusion [47]. The purpose of pathogen-induced cell extrusion is difficult to ascertain, particularly since it can have benefits for both the pathogen and the host. For *Salmonella*, intestinal epithelial cell extrusion has been shown to promote the pathogen's spread within, and escape from, the intestinal tract [49]. Cell extrusion could also promote the egress of *V. parahaemolyticus* from the intestine since extruded cells and debris often had adherent *V. parahaemolyticus*. However, it is also possible that extrusion aids the host, by enabling shedding of adherent bacteria. In support of this idea, it has been found that several bacterial pathogens (e.g., *Shigella*, *Neisseria*) produce factors that appear to counteract epithelial shedding [50].

Some of the ultrastructural changes *V. parahaemolyticus* elicits in intestinal epithelial cells are reminiscent of phenotypes previously described for EPEC, a member of the A/E family of pathogens [31], [32]. Similar to EPEC-induced changes in small intestine explants, we observed long spaghetti-like protrusions from epithelial cells surrounding the edges of the *V. parahaemolyticus* clusters, effacement of microvilli, and the close apposition of individual *V. parahaemolyticus* cells to the effaced epithelial surface within cup-like structures (see Figure 3E). The mechanism(s) that mediate these dramatic alterations in host epithelial cell morphology remain to be determined. However, it seems likely that, similar to EPEC, the activities of some of the effectors translocated by one or both of the *V. parahaemolyticus* T3SS manipulate the host cytoskeleton and thereby alter cell morphology. Indeed, several type III translocated proteins of *V. parahaemolyticus* and the related pathogen *V. cholerae*, including the recently described VopV, have been shown to alter actin dynamics in cultured cells [51]–[55]. Furthermore, AM-19226, a non-O1, non-O139 *V. cholerae* strain that encodes a T3SS similar to the *V. parahaemolyticus* T3SS2 causes villus destruction in the small intestine of infant rabbits, suggesting that common effectors translocated by these systems may contribute to the pathology [27]. However, the steps leading to the destruction of intestinal villi by AM-19226 have not been elucidated, and the pathologic features of AM-19226-induced disease differ from those caused by *V. parahaemolyticus*.

Besides damaging the villous epithelium in the small intestine, *V. parahaemolyticus* also causes elevated proliferation of cells in the crypts. Several other enteric pathogens, as well as the microbiota, have been reported to alter intestinal epithelial dynamics [50]. In some cases, pathogen-induced changes in epithelial homeostasis are thought to promote bacterial colonization. Similarly, since *V. parahaemolyticus* damages the epithelium, promoting epithelial renewal could enhance colonization. However, the increased cell proliferation occurs early during *V. parahaemolyticus* infection, suggesting that proliferation is not a direct response to epithelial damage. Identification of the *V. parahaemolyticus* factor(s) that lead to elevated proliferation, and the manner by which they are antagonized by TDH, may yield insight into mechanisms that normally govern turnover of intestinal stem cells.

Our findings suggest that each of the three previously proposed *V. parahaemolyticus* virulence-linked loci – TDH and the two T3SSs – modulate the organism's pathogenicity. Our results confirm and extend earlier ileal-loop-based studies [19], [22] indicating that T3SS2 is the major virulence factor contributing to *V. parahaemolyticus'* enterotoxicity. We observed that T3SS2 is not only required for intestinal fluid accumulation, but it is also essential for colonization of the small intestine. It will be interesting to explore how T3SS2 promotes colonization and investigate if one (or more) translocated effector(s) act in a similar fashion as the EPEC/EHEC translocated intimin receptor, Tir [56], to enable *V. parahaemolyticus* adherence. Alternatively, do the T3SS2 effectors modulate host cell processes, including those of the immune response, to generate a niche permissive for *V. parahaemolyticus* proliferation? For example, increased access to nutrients may occur as a consequence of epithelial disruption.

Unexpectedly, we found that TDH negatively impacts colonization of the upper regions of the small intestine and appears to dampen some aspects of *V. parahaemolyticus*-induced disease. This result contrasts with findings from studies using ligated ileal loops [22], [57], where TDH was found to contribute to fluid accumulation. Differences between the experimental systems may explain these contradictory results; ligated ileal loops are closed systems where intestinal peristalsis is reduced and infections are of limited duration (typically 18 hr).

Collectively, our findings suggest that infant rabbits will be a very useful experimental model to shed light on the pathogen and host factors, and mechanisms that explain the pathogenesis of *V. parahaemolyticus*-induced intestinal disease. It is important to note however, that while many of the features of *V. parahaemolyticus*-induced disease resemble those reported in humans, rabbits do not exhibit all the signs of *V. parahaemolyticus* infection that have been reported. For example, infected individuals can have occult blood in their stool and occasionally present with grossly bloody stools [5], [58]. The presence of blood in the stool appears to correlate with epithelial damage consisting of ‘superficial ulcerations’ in the lower intestine of patients [5], [58]; no pathology was detected within the colons of infected rabbits and neither gross nor occult blood was observed in fecal material obtained from the rabbits. Nevertheless, infant rabbits reproduce the inflammatory enteritis and watery diarrhea that are the chief signs of disease in most infected individuals. Thus, studies using this model host should enable dissection of the complex interplay of pathogen and host factors that result in disease as well as testing of new therapeutics to combat and/or prevent infection.

## Materials and Methods

### Bacterial strains and media

The pandemic *V. parahaemolyticus* isolate RMID2210633 (serotype O3:K6) was used as the wild type in this study. Derivatives of RMID2210633 containing deletions in TDH (Δ*tdhAS*), T3SS1 (Δ*vscN1*), T3SS2 (Δ*vscN2*), TDH and T3SS1 (aka POR-2 (Δ*tdhAS* Δ*vcrD1*)), or all 3 virulence factors (*aka* the triple mutant or POR-3 (Δ*tdhAS* Δ*vcrD1* Δ*vcrD2*) have been previously described [22]. A high copy number plasmid which is stably maintained in vitro and in vivo without selection [26], [59], was used to introduce green fluorescent protein (GFP) constitutively expressed from the lac promoter, into wild type *V. parahaemolyticus*. All strains were routinely grown in LB medium or on LB agar plates containing the appropriate antibiotics at the following concentrations: 50 µg/mL carbenicillin and 50 µg/mL spectinomycin.

### Ethics statement

All animal studies were carried out in accordance with the recommendations in the Guide for the Care and Use of Laboratory Animals of the National Institutes of Health (8^th^ Edition) and the Animal Welfare Act of the United States Department of Agriculture. All protocols were reviewed and approved by the Harvard Medical Area Standing Committee on Animals (Animal Welfare Assurance of Compliance #A3431-01).

### Animals

Litters of two-day old New Zealand White infant rabbits with the lactating doe were acquired from a commercial breeder (Milbrook Farm, Amherst, MA). The following day, the infant rabbits were administered cimetidine (50 mg kg^−1^ via intraperitoneal injection; Hospira, IL) 3 hr prior to oro-gastric inoculation with either 1×10^9^ cfu wild type *V. parahaemolyticus*, or one of the isogenic mutants, or sodium bicarbonate solution (2.5 g in 100 mL; pH 9) using a size 4 French catheter (Arrow International, Reading, PA). To prepare the inocula, cultures of bacteria grown for ∼18 hr at 30°C were harvested by centrifugation (5 mins 6000 g), and the cell pellet resuspended in sodium bicarbonate solution (pH 9) to a final concentration of 2×10^9^ cfu mL^−1^. Following inoculation, the infant rabbits were monitored frequently for clinical signs of illness. Disease was scored at euthanasia as follows: no gross disease (no adherent fecal material on fur and intestines appear normal), intestinal fluid (no adherent fecal material on fur but intestines appeared red, swollen and contained fluid), diarrhea (liquid fecal material stains or adheres to fur, and intestines appeared red, swollen and contained fluid). In most experiments, rabbits were euthanized at fixed times after infection (i.e. 12, 18, 28 or 38 hr PI), but rabbits were euthanized prior to these time points if they appeared moribund (categorized as ‘dead’ in Table 1 and Figure 1A). At necropsy, the intestinal tract from the duodenum to the rectum was removed and processed for microbiological, microscopic and histologic analyses. For some rabbits, the internal organs including the gall bladder, spleen and liver were also collected, homogenized and plated on selective media to check for systemic spread of *V. parahaemolyticus*.

To determine fluid accumulation ratios (FARs), an approx. 5 cm length of the distal small intestine was isolated from the rest of the intestine using silk ligatures. The intestinal section was weighed and then cut every 0.5 cm to release any luminal fluid, and the tissue pieces reweighed. The FAR was calculated as the weight of fluid divided by the weight of the drained tissue. The electrolyte and protein concentrations in serum and diarrheal fluid collected from the ceca of infected rabbits were measured on an Olympus Analyzer (AU-2700) at the Brigham and Woman's Hospital clinical laboratory.

The number of *V. parahaemolyticus* cfu in tissue samples taken from the small and large intestine, cecum and stool were determined after homogenization, serial dilution and plating on LB media containing 50 µg mL^−1^ carbenicillin as described previously [24]. For unknown reasons, rabbits were occasionally not colonized by the pathogen i.e., no *V. parahaemolyticus* cfu were detected in any tissue sample. These rabbits (less than 10%, regardless of strain tested) were excluded from all further analyses. However, any rabbits that contained detectable numbers of *V. parahaemolyticus* cfu in at least one tissue sample were included; for these rabbits, the lower limit of detection was reported for sections where no colonies were detected at the lowest dilution plated, and this value was used in calculation of mean cfu.

For routine histological analyses, tissue segments were fixed in 10% neutral buffered formalin, processed for paraffin embedding and stained with hematoxylin and eosin (H&E). The slides were semi-quantitatively assessed for infiltration of inflammatory cells (heterophils), cell proliferation, and tissue damage by a pathologist blinded to the origin of the tissue. Each histological parameter was evaluated on a 0–4 scale as follows: 0 (normal), 1 (mild), 2 (moderate), 3 (severe) and 4 (severe and extensive).

### RT-PCR assays

Intestinal samples for quantitative real-time PCR assays were prepared as described previously [26]. The sequences for the primers used to detect rabbit IL-6, IL-8, TNF-α, IL-β and GADPH are available upon request. GADPH was used as a control, and all cytokine transcripts were normalized to GADPH using the ΔΔCT method as previously described [60].

### Immunofluorescence studies

Tissue samples used in immunofluorescence studies were briefly fixed in 4% paraformaldehyde and processed as described previously [24], [26]. All sections were first blocked with 5% bovine serum albumin (BSA) in phosphate buffered saline (PBS) for 30–60 min prior to incubation with the appropriate primary antibody (in PBS containing 0.5% BSA). The following antibodies/reagents were used: mouse anti-ZO-1 monoclonal antibody (1/200; #339100, Invitrogen, CA), mouse anti-occludin-1 monoclonal antibody (1/200; #33–1500, Invitrogen, CA), mouse anti-claudin-1 monoclonal antibody (1/500; #37–4900, Invitrogen, CA), mouse anti-macrophage monoclonal antibody (1/200, #MCA874GA, AbD Serotec, UK), or Alexa Fluor 633 phalloidin (1/100; A22284; Invitrogen, OR), usually incubated overnight at 4°C in the dark. After washing in PBS containing 0.5% Tween20 (PBS_T_), the slides were incubated for 1 hr with goat anti-mouse Alexa fluor 546 (1/200; A11030; Invitrogen, CA) as the detection antibody. After further washing, all slides were counterstained with DAPI (1 µg mL^−1^) for 5 mins, rinsed in PBS_T_ and covered with Prolong Gold Antifade mounting media (P36930, Invitrogen, CA). All slides were examined for fluorescence using a Zeiss LSM510 Meta upright confocal microscope, and images were taken with the LSM510 software.

To determine the zone of proliferating cells in the intestine of infected and mock infected rabbits, tissue sections were incubated with mouse anti-Ki67 monoclonal antibody (1/200, #AB8191, Abcam, MA) overnight at 4°C, washed in PBS_T_ and incubated with goat anti-mouse Alexa fluor 546, phalloidin Alexa fluor 633 and DAPI, and imaged as described above. Using these images, the distance from the base of the zone of actively dividing cells to the top of the zone (the Ki-67-positive region), as well as to the top of the villus (defined by DAPI staining), was measured. Relative proliferation was calculated as the length of the zone of proliferating cells relative to villus length.

TUNEL staining was performed on sections obtained from infected and mock infected rabbits at various times after inoculation to evaluate the extent of apoptosis. Staining was performed according to the manufacturer's instructions (*In situ* cell death detection kit; Roche IN), except that the cryopreserved tissue sections were permeabilized for 5 min and the enzyme mixture was applied for 90 min at 37°C.

### Biotin experiment

Biotin was used as a tracer molecule to determine the integrity of the epithelial barrier as described previously [61], [62]. Briefly, immediately after removal of the entire intestinal tract from mock or infected rabbits, the small and large intestine were separated while maintaining their correct orientation (*ie* proximal vs distal ends). EZ-Link Sulfo-NHS-Biotin (Thermo Scientific, IL) was injected slowly (1–2 min) into the lumen via the open (cut) end of the most distal part of the small intestine or colon. After 3 min, the tissue section just proximal to the site of injection was removed, fixed in 4% paraformaldehyde and processed for immunofluorescence staining as described above. Tissue sections were incubated with streptavidin linked to Alexa 546 (1/500; S11225; Invitrogen, CA) for 1 hr at room temperature, before being counterstained with phalloidin Alexa fluor 633 and DAPI as described above. Ileal and colonic tissue from the ileum and colon of control and infected rabbits that were not treated with biotin, exhibited no endogenous biotin activity when incubated with streptavidin only (data not shown).

### Electron microscopy

Intestinal samples were prepared for scanning or transmission electron microscopy as described previously [24], [25]. Samples were examined using a Hitashi S-4800 FESEM 2 kV scanning electron microscope and a JOEL 1200EX -80 kV transmission electron microscope.

### Statistical analyses

The proportion of rabbits with or without diarrhea was compared to wild type using Fisher's exact test. Fluid accumulation ratios and bacterial counts (after log transformation) were statistically analyzed using one way analysis of variance (ANOVA) and Bonferroni's test for multiple comparison. Ratios obtained from Ki67-stained samples were compared using the Student t test assuming unequal variances. Histological scores for infiltration of heterophils, cell proliferation and tissue damage were treated as non-parametric data and statistically analyzed using the Kruskal-Wallis statistic with Dunn's post-test for multiple comparisons. All statistical analyses were performed using GraphPad Prism, San Diego, CA.

## Supporting Information

Figure S1 **Recruitment of macrophages to the distal small intestine of mock or infected rabbits at early and late stages of infection.** Representative immunofluorescence images from mock or *V. parahaemolyticus*-infected rabbits showing macrophage distribution. Rabbits were infected with GFP-expressing *V. parahaemolyticus* (green) and tissues were stained with mouse anti-macrophage antibodies (red), DAPI (blue) to detect nuclei, and phalloidin to stain F-actin (yellow).
(TIF)

Figure S2 **Cell proliferation in the small intestine of mock or** ***V. parahaemolyticus*** **-infected rabbits at 28 hr PI.** Representative immunofluorescence images of small intestinal sections from mock or *V. parahaemolyticus*-infected rabbits stained with anti-Ki67 antibodies to detect actively dividing cells (red), phalloidin to visualize F-actin (yellow) and DAPI to detect DNA (blue).
(TIF)

Figure S3 **Scanning and transmission electron micrographs of the distal small intestine of** ***V. parahaemolyticus*** **- or** ***V. cholerae*** **-infected rabbits.** (A) High magnification image of elongated protrusions surrounding a cluster of *V. parahaemolyticus* adherent to the epithelial surface. Scale bar = 2 µm. (B) High magnification image of elongated microvilli (long arrows) and vesicles (arrowheads) in the distal small intestine of *V. parahaemolyticus*-infected rabbits. Scale bar = 100 nm. (C) Infant rabbits were infected with *V. cholerae* and sections from the distal small intestine were processed for transmission electron microscopy. Bacterial cells were frequently located adjacent to an intact brush border. Scale bar = 1 µm. (D) An example of an elongated *V. parahaemolyticus* cell (marked with an asterisk (*)) in the intestine of *V. parahaemolyticus*-infected rabbits at 28 hr PI. Bacterium is approx. 10 µm long. Scale bar = 2 µm.
(TIF)

Figure S4 **Paracellular permeability is maintained in the colon of** ***V. parahaemolyticus*** **-infected rabbits at 25 hr PI.** Biotin was injected into the colonic lumen of infected rabbits to assess epithelial integrity. Tissues sections were counterstained with phalloidin (yellow) and DAPI (blue) to detect F-actin and nuclei, respectively.
(TIF)

Figure S5 **Intestinal abnormalities in rabbits infected with wild type** ***V. parahaemolyticus*** **, one of the isogenic mutants or following a mock infection.** Representative H&E-stained sections of tissue from rabbits infected with (A) wild type, (B) no bacteria (mock-infected), (C) *tdh* mutant, (D) T3SS1 mutant, (E) T3SS2 mutant and (F) the triple mutant (Δ*tdh* Δ*vcrD*1 Δ*vcrD*2).
(TIF)

Figure S6 **Severe tissue disruption and necrosis in the distal small intestine of rabbits infected with a** ***V. parahaemolyticus*** **TDH mutant.** Representative H&E-stained section of the small intestine showing extensive villi disruption and necrosis at the villi tips. Scale bar = 100 µm.
(TIF)
