# Supplementary figures and images for: Inflammation and Disintegration of Intestinal Villi in an Experimental Model for Vibrio parahaemolyticus-Induced Diarrhea
Source: PLoS Pathog. 2012 Mar 15;8(3):e1002593. doi: 10.1371/journal.ppat.1002593 (PMC3305451; doi:10.1371/journal.ppat.1002593)

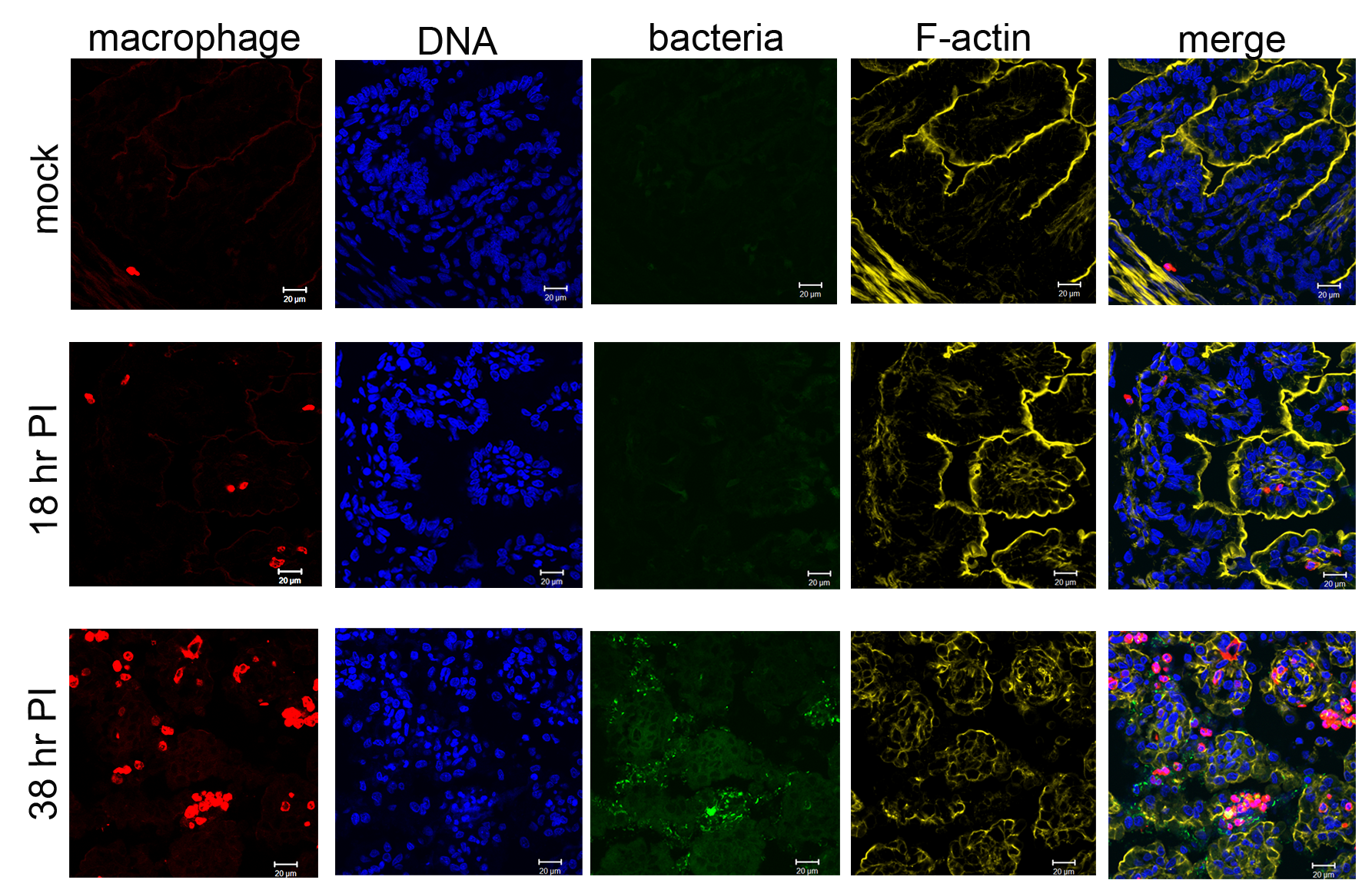

Supplement: Figure S1 — Recruitment of macrophages to the distal small intestine of mock or infected rabbits at early and late stages of infection. Representative immunofluorescence images from mock or V. parahaemolyticus-infected rabbits showing macrophage distribution. Rabbits were infected with GFP-expressing V. parahaemolyticus (green) and tissues were stained with mouse anti-macrophage antibodies (red), DAPI (blue) to detect nuclei, and phalloidin to stain F-actin (yellow). (TIF) [file ppat.1002593.s001.tif]

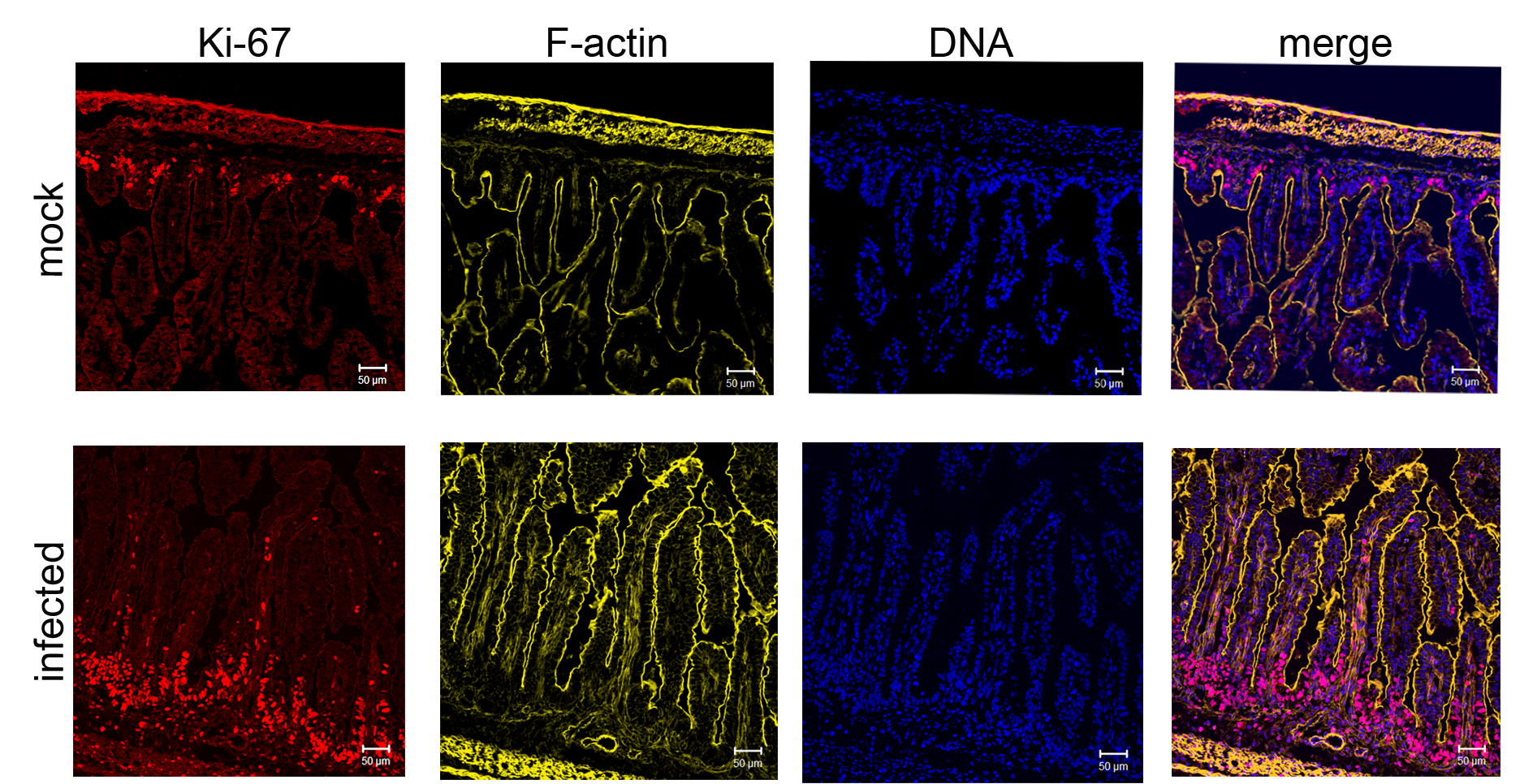

Supplement: Figure S2 — Cell proliferation in the small intestine of mock or V. parahaemolyticus -infected rabbits at 28 hr PI. Representative immunofluorescence images of small intestinal sections from mock or V. parahaemolyticus-infected rabbits stained with anti-Ki67 antibodies to detect actively dividing cells (red), phalloidin to visualize F-actin (yellow) and DAPI to detect DNA (blue). (TIF) [file ppat.1002593.s002.tif]

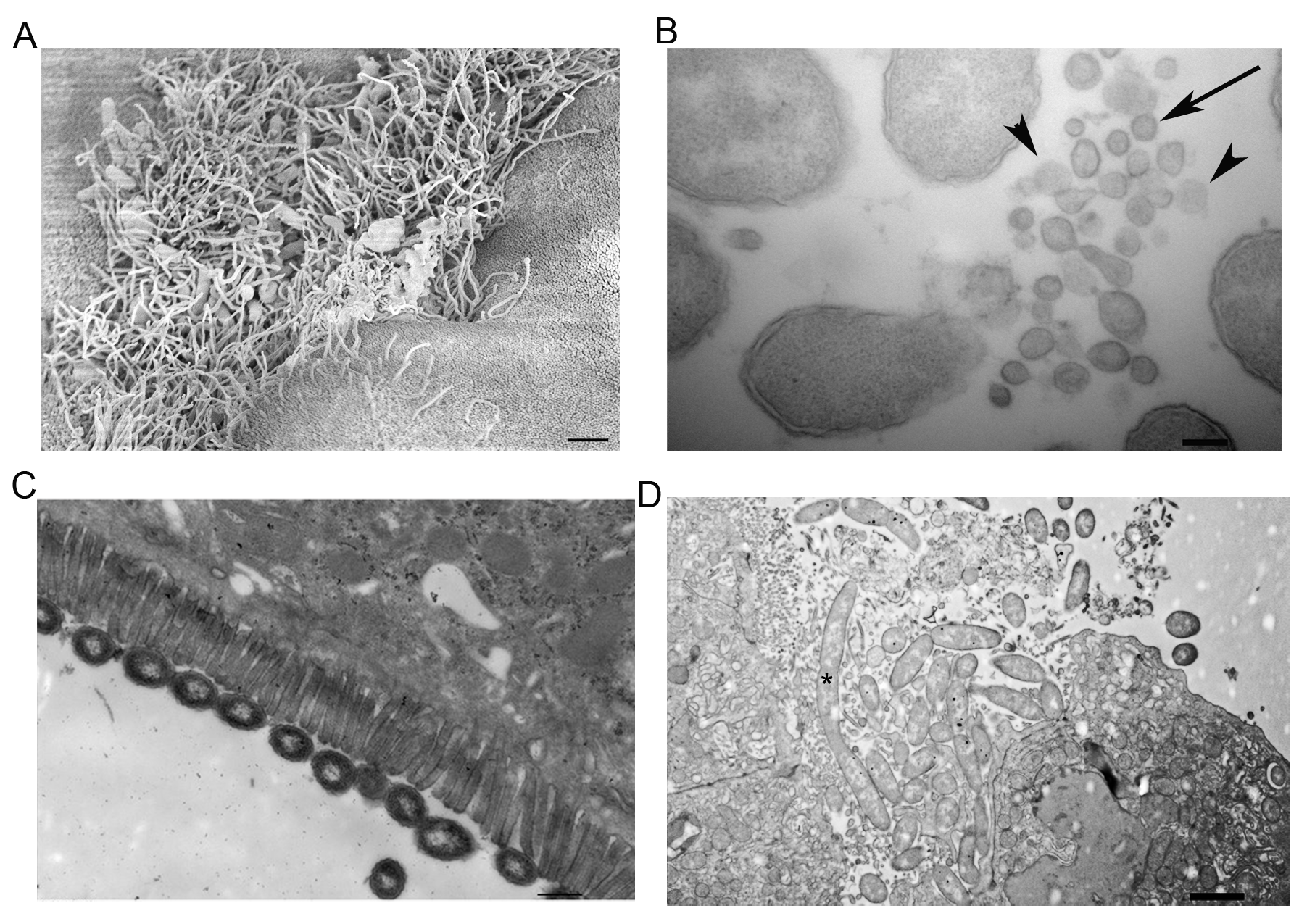

Supplement: Figure S3 — Scanning and transmission electron micrographs of the distal small intestine of V. parahaemolyticus - or V. cholerae -infected rabbits. (A) High magnification image of elongated protrusions surrounding a cluster of V. parahaemolyticus adherent to the epithelial surface. Scale bar = 2 µm. (B) High magnification image of elongated microvilli (long arrows) and vesicles (arrowheads) in the distal small intestine of V. parahaemolyticus-infected rabbits. Scale bar = 100 nm. (C) Infant rabbits were infected with V. cholerae and sections from the distal small intestine were processed for transmission electron microscopy. Bacterial cells were frequently located adjacent to an intact brush border. Scale bar = 1 µm. (D) An example of an elongated V. parahaemolyticus cell (marked with an asterisk (*)) in the intestine of V. parahaemolyticus-infected rabbits at 28 hr PI. Bacterium is approx. 10 µm long. Scale bar = 2 µm. (TIF) [file ppat.1002593.s003.tif]

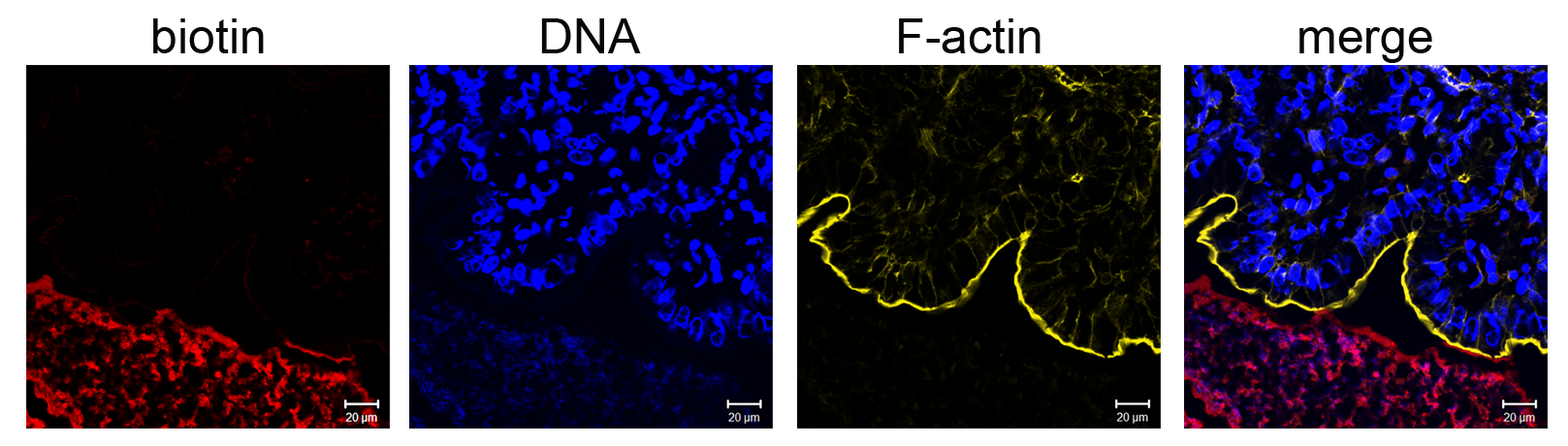

Supplement: Figure S4 — Paracellular permeability is maintained in the colon of V. parahaemolyticus -infected rabbits at 25 hr PI. Biotin was injected into the colonic lumen of infected rabbits to assess epithelial integrity. Tissues sections were counterstained with phalloidin (yellow) and DAPI (blue) to detect F-actin and nuclei, respectively. (TIF) [file ppat.1002593.s004.tif]

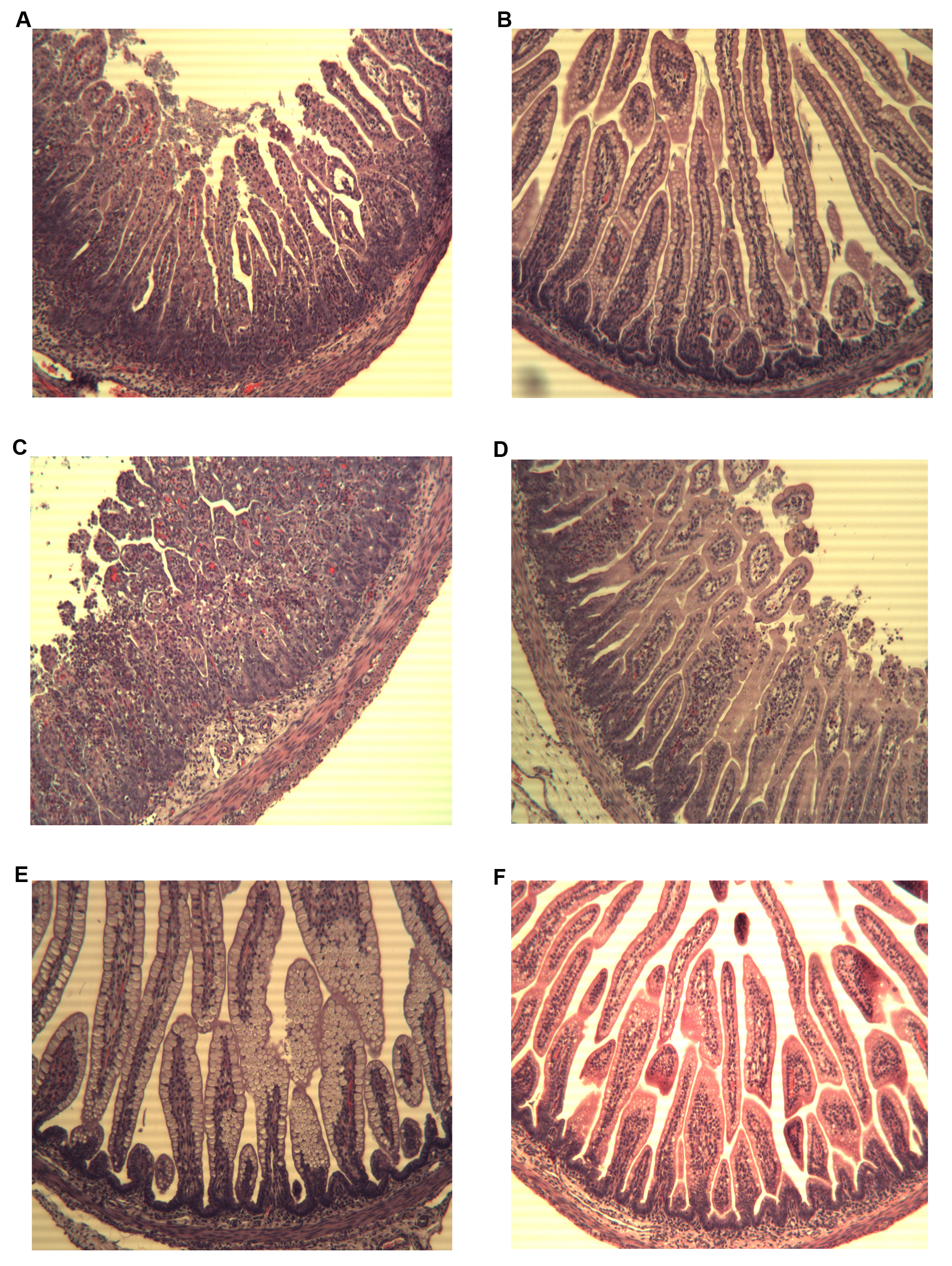

Supplement: Figure S5 — Intestinal abnormalities in rabbits infected with wild type V. parahaemolyticus , one of the isogenic mutants or following a mock infection. Representative H&E-stained sections of tissue from rabbits infected with (A) wild type, (B) no bacteria (mock-infected), (C) tdh mutant, (D) T3SS1 mutant, (E) T3SS2 mutant and (F) the triple mutant (Δtdh ΔvcrD1 ΔvcrD2). (TIF) [file ppat.1002593.s005.tif]

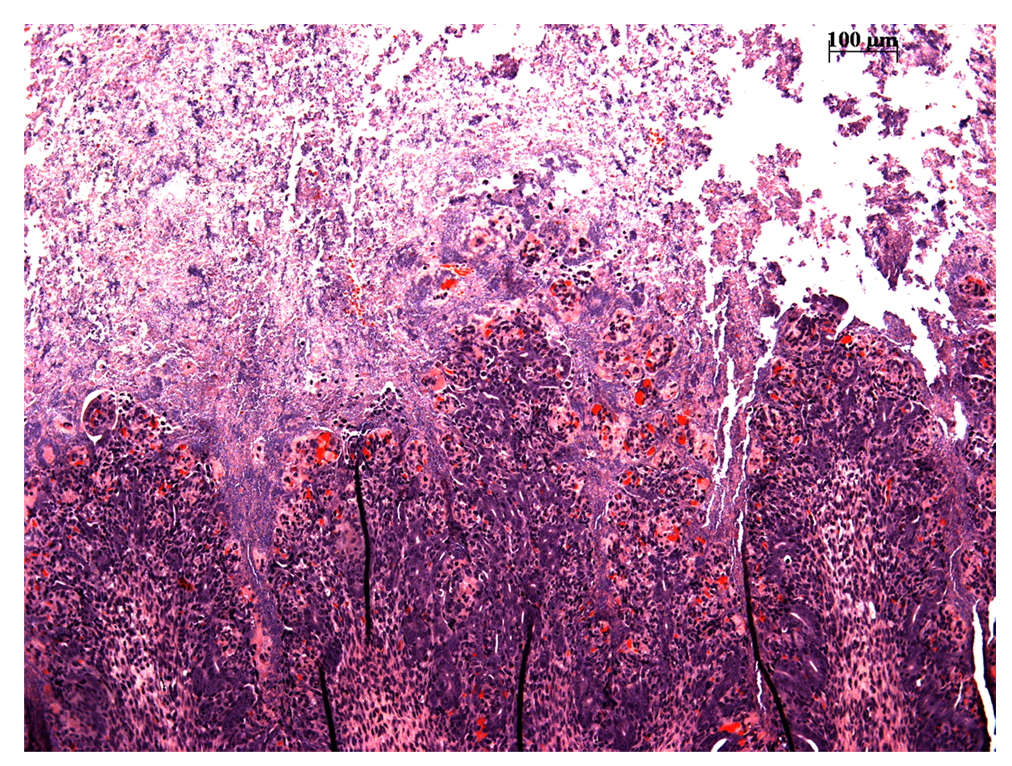

Supplement: Figure S6 — Severe tissue disruption and necrosis in the distal small intestine of rabbits infected with a V. parahaemolyticus TDH mutant. Representative H&E-stained section of the small intestine showing extensive villi disruption and necrosis at the villi tips. Scale bar = 100 µm. (TIF) [file ppat.1002593.s006.tif]
